# Targeting Triple Negative Breast Cancer with a Dinuclear Copper(II) Thiocarbohydrazone Complex: Efficacy Evaluation and Cellular Response

**DOI:** 10.1021/acsomega.5c04277

**Published:** 2025-09-03

**Authors:** Kumudini Paliwal, Abinash Swain, Durga Prasad Mishra, Manjuri Kumar

**Affiliations:** † Department of Chemical Engineering, Birla Institute of Technology and Science Pilani, K.K. Birla Goa Campus, Zuarinagar, Sancoale, Goa 403726, India; ‡ 30082 Cell Death Research Laboratory, Endocrinology Division, CSIR-Central Drug Research Institute, B.S. 10/1, Sector-10, Jankipuram Extension, Lucknow, Uttar Pradesh 226031, India

## Abstract

The importance of copper complexes in bioinorganic chemistry and medicinal chemistry has been thoroughly documented in the literature. In recent years, extensive studies have focused on copper complexes of Schiff bases that can bind to DNA and proteins, exhibiting significant cytotoxic activity against various cancer cell lines, paving the way for the development of novel copper-based anticancer therapeutics. In this regard, we have synthesized a new dinuclear Cu­(II) complex **1** (**K38**) with a thiocarbohydrazone Schiff base ligand in order to study its anticancer potential. This complex was characterized by HRMS, IR, UV–vis, and EPR spectroscopic studies. The methanol frozen glass of the compound at liquid nitrogen temperature (LNT) exhibited an axial EPR spectrum which was found to contain two sets of parallel lines suggesting the presence of two very weakly interacting Cu­(II) centers with average *g*_∥_ = 2.258 and *g*_⊥_ = 2.057 values (approximately) suggesting a pseudo-octahedral or distorted square pyramidal structure of each Cu­(II) center with axial (*Z*) elongation and the unpaired electron being in the d_*x*_^2^–_*y*_^2^ orbital of each of the Cu­(II) ions. Biological studies revealed strong DNA binding affinity of complex **1**, exhibiting an intrinsic binding constant *K*_b_ of 1.14 × 10^5^ M^–1^. Further, **1** was also capable of DNA cleavage and DNA fragmentation as observed by TUNEL positive cells. Cytotoxicity assays indicated potent effects against tested breast cancer (BC) cell lines like MDA-MB-231 and MCF-7 where the IC_50_ values were found to be 0.41 ± 0.03 and 0.89 ± 0.08 μM, respectively. On the other hand, compound **1** was found to be remarkably nontoxic in the nonmalignant breast epithelial MCF-10A cells even at very high concentrations of **1** (75 μM) showing its selectivity toward TNBC (MDA-MB-231) cells. Complex **1** generated substantial ROS as evident from the DCFDA assay. Mechanistic studies using western blot showed a caspase cascade triggered by complex **1** through an intrinsic mitochondrial pathway. Complex **1** led to a decrease in mitochondrial mass, increased mROS, and significant mitochondrial membrane depolarization as evident from MitoTracker, MitoSOX, and TMRM assays, confirming mitochondrial dysfunction leading to cell death.

## Graphical abstract

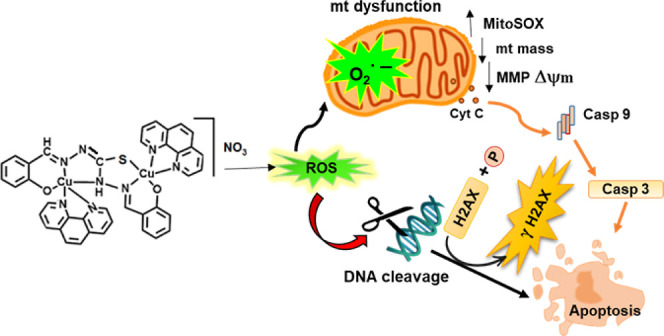

## 1 Introduction

Metal coordination complexes have been extensively studied, driven by their potent biological activities and diverse functional properties.^1–7^ Some of these metal complexes are reported to show significant antitumor potential applicable in chemotherapy for treating various cancers.^8–11^ Cisplatin is one such metal complex that is the most potent anticancer drug; however, it faces limitations due to severe toxicity and the development of drug resistance. In recent years, there has been considerable growth in the research and development of new metal-based anticancer drugs aimed at enhancing clinical efficacy, reducing toxicity, and expanding their range of activity.^12^ Among nonplatinum metal compounds, copper complexes are particularly promising as anticancer agents, with the potential for reduced toxicity due to the presence of endogenous metal.^13^

Copper is an essential trace element as it plays a vital role in various physiological and biological functions like cell respiration, proliferation, enzyme transport, and immune regulation. It is known that copper has the potential to form complexes by coordination to different ligand systems and can act as a potential drug for cancer treatment. One important aspect for the cytotoxicity of copper complexes is due to their redox activity that can generate reactive oxygen species (ROS) in higher concentrations that can cause DNA damage, leading to cell death. Literature suggests that the copper complex can inhibit growth and division of cells which may lead to apoptosis in cancer cells.^14–16^ In addition, these complexes have the potential to regulate metastasis and angiogenesis and help the immune system kill cancer cells. Given these promising effects, exploring versatile ligand classes becomes essential in developing effective therapeutic agents.^17^

Schiff bases are one such popular class of ligands in medicinal chemistry that are recognized for their pharmaceutical applications. This ligand system is characterized by the presence of an imine group (C=N) and synthesized through the condensation of a primary amine, aliphatic, or aromatic (R–NH_2_ or Ar–NH_2_), with an active carbonyl compound, such as an aldehyde or ketone. Schiff bases are popular in coordination chemistry due to their ease of synthesis, versatility, and favorable electronic properties. They readily form stable complexes with a wide range of metal ions through the imine (>C=N−) nitrogen. Schiff base ligands are considered as “privileged ligands” because they efficiently stabilize complexes of main-group, transition, lanthanide, and actinide elements. Their coordination chemistry has found extensive applications in catalysis, analytical chemistry, metallurgy, and bioinorganic research.^18^ Many derivatives of Schiff base ligands like semicarbazone, thiosemicarbazone, carbohydrazone, and thiocarbohydrazone compounds have attracted the attention of researchers due to their broad biological properties.^19,20^ The advantage of thiocarbohydrazone over thiosemicarbazones for metal-based drug development is primarily due to the presence of extra metal binding motifs such that they can be used to synthesize both mononuclear as well as dinuclear metal complexes. Thiocarbohydrazone has shown antibacterial, antifungal, enzyme inhibition as well as anticancer activity.^20–22^ Despite the potential, there are limited reports on the biological activity of their metal complexes.^23–25^

In this work, 1,5-bis­(salicylidene)­thiocarbohydrazide as the primary ligand and 1,10-phenanthroline as the secondary ligand are coordinated with copper. The main ligand comprises N, S, and O donors which hold a lot of significance in the field of chemistry as they exhibit biological relevance, good chelation, and variable coordination. This supports formation of complexes with different metals and increases their value in applied research.^26^ The current study focuses on DNA binding and plasmid DNA cleavage properties of the synthesized copper complex. Additionally, the mechanism of DNA damage, resulting in cell cycle arrest and cell death, is also reported, focusing on the role of mitochondrial dysfunction leading to apoptotic cell death.

## 2 Results and Discussion

### 2.1 Synthesis and Characterization

Schiff base 1,5-bis­(salicylidene)­thiocarbohydrazide exists in its thioketo form (A) in the solid^27^ as evident from the presence of ν­(C=S) band at 1280 cm^–1^ in the IR spectrum of the free Schiff base ligand (vide infra), however, in solution it coexists with its thiol form (syn or anti) as reflected in Scheme 1(B or B′). This compartmental ligand has two distinct coordination sites, the first site of the enolized ligand as shown in the antiform (B′) comprising thiolate S^–^, phenolate O^–^, and the imine N; while the second site comprises N of NH, N of imine, and the remaining phenolate O^–^, behaving as two tridentate binding sites for the metal ion with overall three negative charges. Thus, when cupric nitrate (2 mmol) is reacted with this Schiff base ligand (1 mmol) in the presence of 1, 10-phenanthroline (2 mmol), both sites are occupied by Cu­(II) ions each having a five-coordinate geometry as shown in Scheme 1, thus forming a dinuclear monocationic complex. The overall positive charge on the complex is neutralized by the counteranion NO_3_^–^ and is confirmed from the measurement of conductivity of the compound in DMF (Λ = 74.9 Ohm^–1^ cm^2^ mol^–1^) where it acts as a 1:1 electrolyte.^28^ This strongly suggests that the Schiff base ligand is acting as a trianionic ligand in the present complex **1** (**K38**) as shown in Scheme 1.

**Scheme 1:**
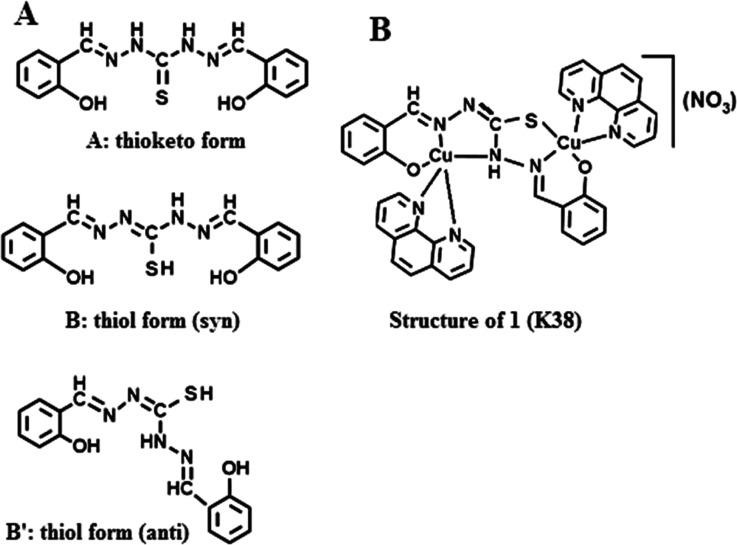
(A) Schiff Base Ligand 1,5-bis­(salicylidene)­thiocarbohydrazide in its *thioketo*- and *thiol*- Forms and (B) the Proposed Structure of the Dinuclear Cu­(II) Complex **1** Formed

### 2.2 HRMS Spectra

The positive-ion mode HRMS spectra of complex **1 (K38)** were recorded in CH_3_OH. The spectra are shown in Figure S1 (Supporting Information) and the results with assignments are presented in Table 1 below.

**Table 1:** HRMS Spectral Results for K38 in Methanol

HRMS peak (*m*/*z*)			
observed at	calculated	calculated for^a^	corresponding to
504.09	504.31	[Cu_2_ (HL)^3–^(CH_3_OH)_2_ + H]^+^	[Cu_2_ (C_15_H_11_N_4_O_2_S) (CH_3_OH)_2_ + H]^+^
624.67	624.43	[(Cu_2_((HL)^3–^ (sal)^−^ (CH_3_OH)_2_ + H]^+^	Cu_2_(C_15_H_11_N_4_O_2_S)(C_7_H_5_O_2_) (CH_3_OH)_2_ + H]^+^
711.61	711.48	[(*o*-phen)Cu_2_(H L′)^2–^ (NO_3_)^−^ (CH_3_OH)]^+^	[(C_12_H_8_N_2_) Cu_2_(C_15_H_10_N_4_O_2_S) (NO_3_) ^–^ (CH_3_OH)]^+^
739.65	739.64	[(*o*-phen)Cu_2_ (HL)^3–^(sal)^−^]^+^	[(C_12_H_8_N_2_) Cu_2_ (C_15_H_11_N_4_O_2_S) (C_7_H_5_O_2_)]^+^
750.44	750.48	[(*o*-phen)Cu_2_(H L′)^2–^ (CH_3_OH)(NO_3_)^−^ + K]^+^	[(C_12_H_8_N_2_) Cu_2_(C_15_H_10_N_4_O_2_S) (CH_3_OH)(NO_3_) ^–^ + K]^+^
760.12	759.64	[(*o*-phen)Cu_2_(H_2_L)^2–^ (sal)^−^ (H_2_O) + H)]^+^	[(C_12_H_8_N_2_) Cu_2_ (C_15_H_12_N_4_O_2_S) (C_7_H_5_O_2_) (H_2_O) + H]^+^
795.53	795.60	[(*o*-phen)Cu_2_ (H L′)^2–^ (sal)^−^ (H_2_O)+ K]^+^	[(C_12_H_8_N_2_) Cu_2_(C_15_H_10_N_4_O_2_S) (C_7_H_5_O_2_) (H_2_O)+ K]^+^
864.13	863.73	[(*o*-phen)_2_ Cu_2_(HL)^3–^ (CH_3_OH)_2_ + H]^+^	[(C_12_H_8_N_2_)_2_ Cu_2_ (C_15_H_11_N_4_O_2_S) (CH_3_OH)_2_ + H]^+^
889.49	889.82	[(*o*-phen)_2_ Cu_2_(HL)^3–^ (TSC)]^+^	[(C_12_H_8_N_2_)_2_ Cu_2_ (C_15_H_11_N_4_O_2_S) (CH_5_N_3_S)]^+^
915.98	915.69	[(*o*-phen)_2_Cu_2_(HL)^3–^ (NO_3_)^−^ (H_2_O)_3_ + H]^+^	[M+3H_2_O + H]^+^ or [(C_12_H_8_N_2_)_2_ Cu_2_ (C_15_H_11_N_4_O_2_S) (NO_3_)^−^ (H_2_O)_3_ + H]^+^

a Abbreviations used in Table 1 and the chemical and structural formulas of the species directly or indirectly observed in the HRMS spectra are given in Schemes S1 and S2 of the Supporting Information.

### 2.3 IR Spectra

The free/unbound ligand 1,5-bis­(salicylidene)­thiocarbohydrazide shows a medium intensity IR band around 3140 cm^–1^ (Figure S2A) arising due to ν­(N–H). The ν­(C=N) band appears as a strong band at 1612 cm^–1^ in the IR spectrum of the unbound ligand (Figure S2A) which is observed to be shifted to 1599 cm^–1^ upon complex formation, suggesting imine nitrogen (C=N) coordination to the metal ion (Cu^2+^) in complex **1**. The ν­(C=S) band at 1280 cm^–1^ in the IR spectrum of the free ligand is absent in complex **K38**, suggesting that the ligand in its thiol form coordinated to Cu^2+^. The absence of the ν­(O–H) in the complex **K38** IR spectrum suggests O–H group deprotonation and coordination of two phenolate O^–^ in the complex, making it a trianionic ligand consisting of two Cu^2+^ ions (one ion on each side) such that, there is an overall positive charge on the complex. This suggests that the complex can accommodate one counteranion like NO_3_^–^ as Cu­(NO_3_)_2_·3H_2_O is used during the synthesis of complex **K38**. Further, a band near 1300 cm^–1^ in the IR spectrum of the complex is observed indicating the presence of the NO_3_^–^ moiety, this band is not observed in the free/unbound ligand (Figure S2C). It is known from the literature that a very strong IR spectrum band centered around 1370 cm^–1^ indicates the presence of cupric nitrate.^29,30^ The presence of the NO_3_^–^ moiety is further supported by the HRMS results of the complex (presented above).

### 2.4 Electronic Spectra

The green methanol solution of the compound showed a broad band (Figure S3 inset) centered around 640 nm (ε = 311 mol^–1^·cm^–1^) originating from the ligand field (d–d) transitions. Additionally, one peak was observed at 270 nm (ε = 7401.5 mol^–1^·cm^–1^) with three distinct shoulders at 400 (ε = 1192 mol^–1^·cm^–1^), 330 (ε = 2343 mol^–1^·cm^–1^), and 285 nm (ε = 4961.5 mol^–1^·cm^–1^), respectively, due to charge transfer transitions. This compound was found to be highly stable in Tris–HCl buffer (pH 7.44) as well as in DMEM medium, as revealed from the electronic spectra (Figure S4) recorded with time (negligible decrease in absorbance after standing for 48 h). However, all experiments were carried out always with freshly prepared solution of the compound.

### 2.5 EPR Spectra

Strong X-band EPR spectra exhibited by compound **1** in its powder state as well as in solution (Figure 1) confirmed that it is paramagnetic. The compound displayed an isotropic RT powder spectrum (Figure 1A), and the *g* value was found to be ∼2.099. On the other hand, the compound exhibited a spectrum in methanol at RT (Figure 1B) that is neither a four-line spectrum (^63/65^Cu nucleus, *I* = 3/2) nor a seven-line spectrum. However, the methanol frozen glass at liquid nitrogen temperature (LNT) exhibited an axial EPR spectrum (Figure 1C) which was found to contain two sets of parallel lines suggesting the presence of two very weakly interacting Cu­(II) centers with average *g*_∥_ = 2.258 and *g*_⊥_ = 2.057 values (approximately) suggesting a pseudo-octahedral or distorted square pyramidal structure of each Cu­(II) center with axial (*Z*) elongation and the unpaired electron being in the d_*x*_^2^–_*y*_^2^ orbital of each of the Cu­(II) ions. Moreover, the central line of the frozen glass spectrum (Figure 1D) clearly showed 7 superhyperfine lines possibly originating from the interaction with the nuclei of the three N (*I* = 1) atoms in the equatorial plane that are coordinated to the Cu­(II) ion in the second site. The experimental parameters are provided in Figure S5A,D of the Supporting Information with each individual EPR spectrum.

**Figure 1:**
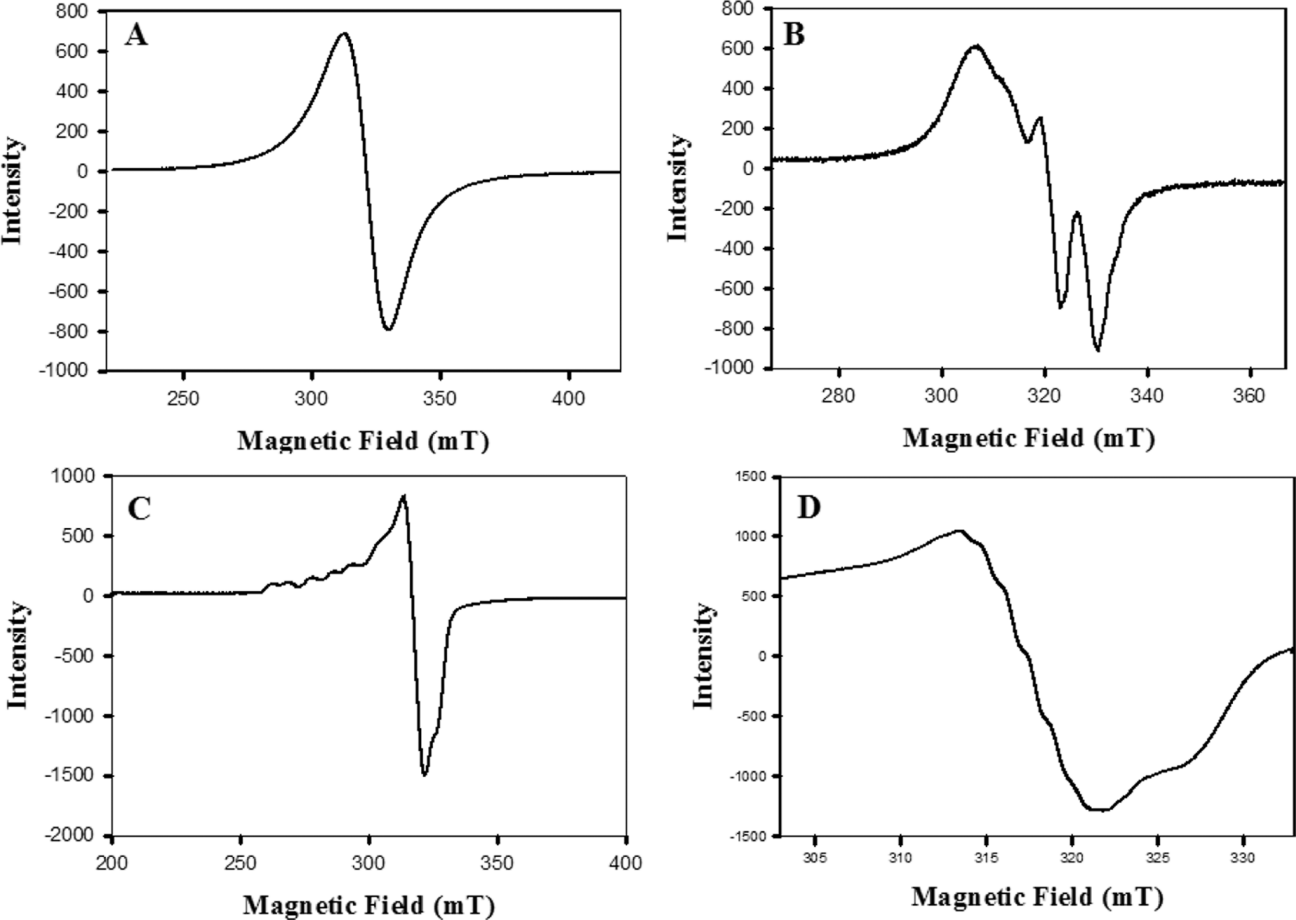
X-band EPR spectra of **1**: (A) powder at RT, (B) in methanol solution at RT, (C) methanol frozen glass at LNT, and (D) central line of the LNT spectrum showing the *N*-hyperfine lines. Other experimental parameters are provided in the Supporting Information (Figure S5A,D) with each individual EPR spectrum.

### 2.6 Interaction with DNA

#### 2.6.1 Absorbance Titration

It is a common technique employed to understand the binding behavior of small molecules with some macromolecules, like DNA.^31–33^ Here, calf thymus DNA dissolved in Tris–HCl was used to study the interaction of complex **1** with DNA. Binding of complexes to DNA leads to the perturbation of absorption bands. If the absorption intensities of drugs are decreased (hypochromism) upon increasing the concentration of CT-DNA, it indicates drug binding with DNA via intercalation, while if the absorption intensities of drugs are increased (hyperchromism) it indicates partial or nonintercalative mode of binding, like electrostatic forces, van der Waals interaction, hydrogen bonds, and hydrophobic interaction. The spectral changes as observed from Figure 2A show a 34% decrease in intensity of absorbance for complex **1**, after addition of increasing amounts of calf thymus DNA. This hypochromism indicates toward intercalative mode of DNA binding, wherein the ligands stack between the base pairs of the macromolecule, i.e., DNA in this case, leading to lengthening and unwinding of DNA strands.^34^ In order to get a quantitative analysis of this intercalative binding, the intrinsic binding constant *K*_b_ value for complex **1** was calculated using the Wolfe–Shimmer eq (eq 1).^35^

1 $$\frac{\left[DNA\right]}{\left(\varepsilon_{a}-\varepsilon_{f}\right)}=\frac{\left[DNA\right]}{\left(\varepsilon_{b}-\varepsilon_{f}\right)}+\frac{1}{K_{b}\left(\varepsilon_{b}-\varepsilon_{f}\right)}$$

where, the concentration of CT-DNA is represented by [DNA] and extinction coefficients of the free, apparent, and bound metal complex are represented by ε_f,_ ε_a_, and ε_b_, respectively.

**Figure 2:**
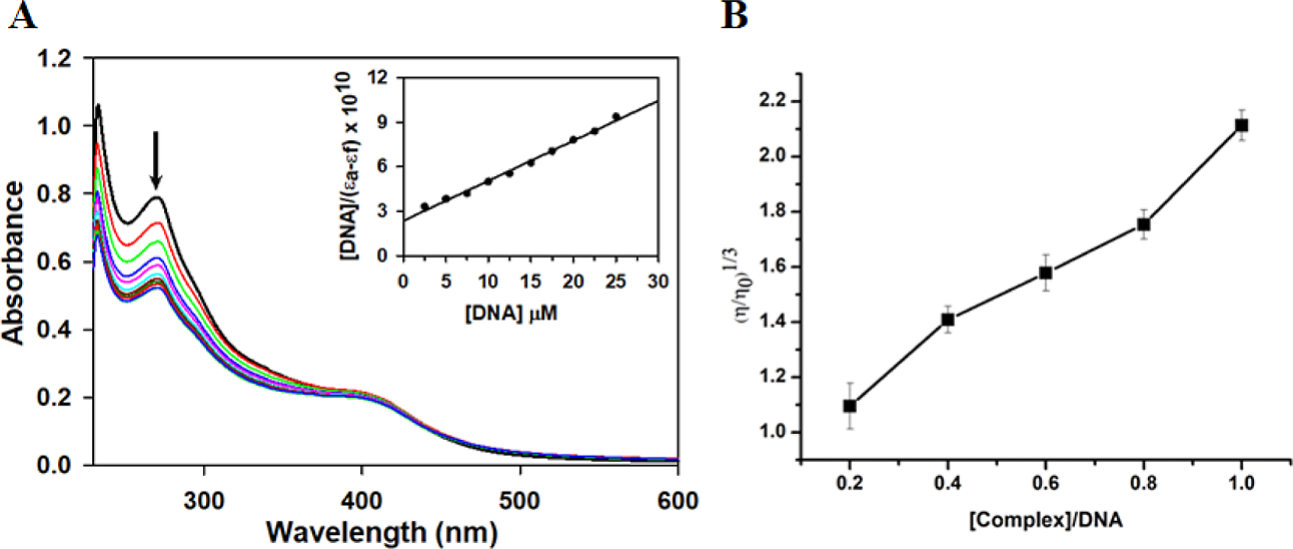
(A) Changes in absorbance spectra of **1** (1 × 10^–5^ M) in Tris–HCl solution (pH 7.4), with the increasing concentration of CT-DNA (0–25 μM). Inset shows plot of [DNA]/(ε_a_ – ε_f_) versus [DNA], (*R* ^2^ = 0.99627 for 10 points). (B) The graph showing relative specific viscosity (η/η_0_)^1/3^ versus [complex]/[DNA].

In order to calculate the *K*_b_ value for complex **1**, the ratio of slope to intercept from the plot of [DNA]/(ε_a_ – ε_f_) versus [DNA] was used. The value of *K*_b_ was found to be 1.14 × 10^5^ M^–1^ which is very close to that of the classical DNA intercalator EB, *K*_b_ = 3.3 × 10^5^ M^–1^.^36^ Thus, it can be inferred that the binding interaction of complex **1** with DNA is very strong as its calculated *K*_b_ value is of the same order as that of EB. It is to be noted that this exceptionally high *K*_b_ value is rarely reported in Cu complexes, emphasizing the importance of this synthesized Cu­(II) complex **1**. This significant hypochromism, along with high *K*_b_ value indicates strong binding of complex **1** to CT-DNA by intercalative mode. These results can be compared with some reported Cu­(II) complexes such as [Cu_2_(L^1^)_2_(py)_4_] and [Cu_2_(L^2^)_2_(py)_4_] having binding constants (*K*_b_) of 10.3 ± 0.02 × 10^4^ M^–1^ and 6.91 ± 0.01 × 10^4^ M^–1^, respectively.^37^ Other examples are [CuL^1^ and [CuL^1^ Py] with binding constants (*K*_b_) of 5.20 × 10^4^ and 1.48 × 10^4^, respectively, reported by Nair and Attokkaran.^38^ Likewise, Mondal et al. reported two copper complexes Cu­(DC)_2_ and Cu (DT)_2_ with *K*_*b*_ values of 3.5 × 10^4^ and 2.2 × 10^4^, respectively.^39^

#### 2.6.2 Viscosity Measurement

Viscosity measurement is one of the most effective methods to confirm the mode of DNA binding as changes in DNA’s structure due to molecule binding, specifically change in length, can be detected through the measurement of viscosity changes. The phenomenon of hypochromism observed during electronic spectral absorbance titration points toward DNA binding via intercalation.^40,41^ However, hydrodynamic methods like viscosity measurement that rely on the change of DNA length are important in order to confirm the mode of DNA binding.^42^ As already discussed, intercalation involves stacking of the complex between the DNA base pairs, which leads to an increase in the length of DNA strands and ultimately causes an increase in the viscosity values. No such effect is reported in the case of groove binding. So, viscosity measurement was performed using complex **1**. Figure 2B shows a plot of relative specific viscosity (η/η_0_)^1/3^ versus [complex]/[DNA], and it is clear from this result that there is an increase in the viscosity with an increment in the concentration of complex, confirming the intercalative mode of DNA binding. Similar results supporting intercalative mode of DNA binding by copper complexes have been reported in the literature.^37^ Furthermore, viscosity changes by addition of cisplatin to CT-DNA were also analyzed and the results obtained are in agreement with the data reported in the literature^43^ for covalent binding as seen from Figure S6 (Supporting Information).

#### 2.6.3 Ethidium Bromide Displacement Assay

This assay is a widely used technique to study the binding of ligands to nucleic acids like DNA. Ethidium bromide (EB) produces significant fluorescence only upon binding to DNA.^44^ When there is binding competition between EB and the metal complex on DNA strands it may lead to displacement of some of this bound EB causing reduction in the fluorescence from the initial level.^21,44,45^ Thus, the experiment allows evaluation of the binding potential of this competing metal complex. Figure 3 shows a decrease in fluorescence intensity when complex **1** was added to a DNA-EB adduct (top black curve). Further, the fluorescence intensity kept decreasing upon increasing the concentration of complex **1**. Thus, it is established that complex **1** can displace EB from binding sites on DNA making it a strong DNA binder.^46^

**Figure 3:**
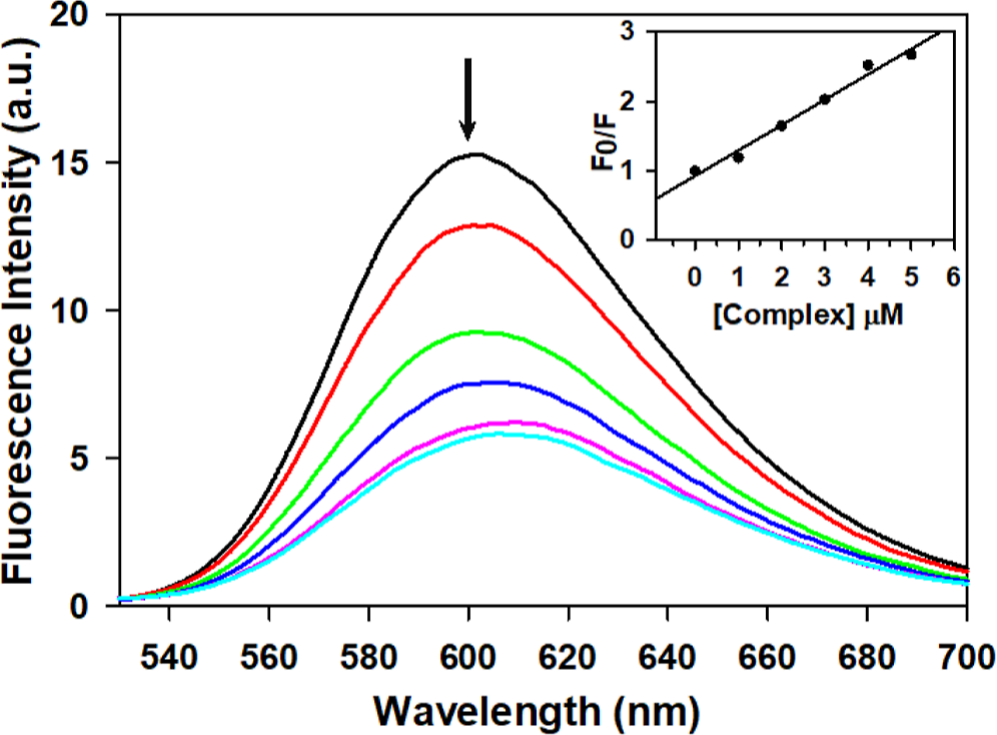
Competitive DNA binding after addition of increasing amount of **1 (K38)** (0 – 5 μM) to the EB-CT DNA adduct. [CT DNA] = 50 μM and [EB] = 2 μM. Inset showing plot of *F*_0_/*F* vs [Complex **1**] (*R* ^2^ = 0.99129).

The Stern–Volmer eq 2 was plotted (inset of Figure 3) and used to calculate the Stern–Volmer quenching constant, *K*_SV_. In eq 2, *F*_0_ and *F* represent fluorescence intensity without quencher and with quencher, respectively, and [*Q*] is the complex concentration which is the quencher.

2 $$\frac{F_{0}}{F}=1+K_{SV}\left[Q\right]$$

The value of *K*_SV_ was calculated from the slope of the plot of *F*_0_/*F* versus [*Q*] and was calculated as 3.6497 × 10^5^ M^–1^ which suggests very strong affinity of complex **1** for DNA. In addition, the apparent binding constant was also determined using eq 3.

3 $$K_{EB}\times \left[EB\right]=K_{app}\times {\left[complex\right]}_{50}$$

Here, [complex]_50_ represents the concentration of the Cu­(II) complex at which the intensity of fluorescence is reduced to 50% of that of the initial EB-DNA adduct. The concentration of EB was taken as 2 μM and the value of *K*_EB_ is 1 × 10^7^ M^–1^. The apparent binding constant was calculated as 1.35 × 10^7^ M^–1^ which is slightly higher than even the binding constant of EB, the classical intercalator. Thus, the binding potential of complex **1** seems to surpass that of EB. These results are comparable with the reported copper complexes [Cu­(DC)_2_] and [Cu (DT)_2_] with an apparent binding constant *K*_app_ = 4.1 × 10^5^ and 3.8 × 10^5^, respectively.^39^ Similarly, Hoque et al. have reported high *K*_sv_ values of 1.78 ± 0.05 × 10^6^ and 4.58 ± 0.04 × 10^6^ M^–1^, respectively, for two binuclear copper­(II) complexes [Cu_2_(L^1^)_2_(Py)_4_] and [Cu_2_(L^2^)_2_(Py)_4_].^37^

#### 2.6.4 Nuclease Activity

Since complex **1** has shown excellent DNA binding property, its nuclease activity was also tested using gel electrophoresis with complex **1**-treated plasmid DNA. The pUC19 plasmid DNA is in a supercoiled state naturally, which travels fast during gel electrophoresis. This DNA may undergo single or double stranded scission in the presence of a metal complex showing nuclease activity. When there is a single stranded scission on one strand it converts into a nicked form that travels very slowly. Further, there can be a linear form which forms when there is double stranded scission and this form travels with intermediated speed and is found between the supercoiled and nicked form.^47–49^ It is reported that metal complexes can bring about DNA cleavage by the oxidative or hydrolytic pathway.^50,51^ The oxidative pathway involves oxidative damage of the nitrogen base or sugar moiety in the presence of an oxidizing agent like H_2_O_2_, while the hydrolytic pathway includes phosphodiester bond hydrolysis without the use of any external agent.^52,53^ The image of gel electrophoresis by **K38** (complex **1)**-treated plasmid DNA is shown in Figure 4A oxidative pathway and (B) hydrolytic pathway. It can be observed that **1** has an excellent oxidative cleavage ability. At just 1 μM concentration (lane 4, Figure 4A), there is 100% conversion to the nicked form. In addition, free radical scavengers like DMSO and NaN_3_ were also used to find the free radicals responsible for nuclease activity. As seen from Figure 4A, there is higher inhibition of DNA cleavage treated with DMSO. It is a scavenger for the hydroxyl radical, which suggests that hydroxyl radicals may have an important role in the progression of oxidative cleavage by the synthesized Cu­(II) complex. No such inhibition is observed in lane 6 which has NaN_3_, which suggests that the role of singlet oxygen is not that prominent in this cleavage activity. In addition to oxidative cleavage, the hydrolytic cleavage capacity of complex **1** was also tested, as shown in Figure 4B. It can be observed that the complex is capable of causing moderate cleavage without the presence of any external agent. At a concentration of 120 μM, there is 88% conversion to the nicked form, making complex **1** better at oxidative cleavage as compared to hydrolytic cleavage.

**Figure 4:**
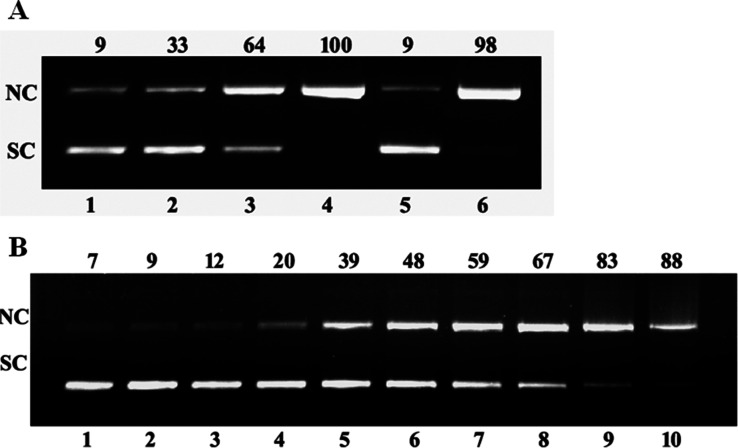
Gel electrophoresis showing nuclease activity on pUC19 plasmid DNA [200 nanogram] by complex **K38** in 50 mM Tris HCl, pH 8 at 37 °C. (A) Lane 1, control - pUC19 as DNA; lane 2–4 [**K38**] = 0.25, 0.5, 1 μM + H_2_O_2_ (1 mM); Lane 5, 1 μM **K38** + 2 μL DMSO; Lane 6, 1 μM **K38** + 500 μM of NaN_3_. (B) Lane 1, pUC19 DNA as control; lane 2–10 [**K38**] = 2.5, 5, 10, 20, 30, 40, 50, 100, 120 μM.

### 2.7 Biological Evaluation/In Vitro Studies

#### 2.7.1 MTT Assay

The cytotoxicity of Cu­(II) complex **K38** on breast cancer (BC) cell lines was determined by using the MTT assay. This assay was used to determine cell viability and depends on the ability of mitochondrial dehydrogenase in viable cells to convert tetrazolium in MTT to insoluble formazan crystals. This purple formazan can be easily detected and indicate the cell viability.^7,54^ In this case, we calculated the half maximal inhibitory concentration IC_50_ by a plot of cell viability versus drug concentration using two breast cancer cell lines MCF-7 and MDA-MB-231 (Figure 5). The IC_50_ values of complex **1** in MCF-7 was 0.89 ± 0.08 μM and that in MDA-MB-231 was 0.41 ± 0.03 μM. These results suggest that the complex has potential to inhibit proliferation in breast cancer cells, especially triple negative breast cancer (TNBC) MDA-MB-231 cells. This TNBC cell line is a particularly aggressive subtype of breast cancer and does not respond well to endocrine therapy or molecular targeted therapy due to its different phenotype.^55^ Chemotherapy is the only treatment option for such cancer, however, development of chemoresistance is a major challenge that leads to treatment failure so development of new anticancer agents is essential.^56^ Further, the MTT assay was also performed using cisplatin and the free ligand 1,5-bis­(salicylidene)­thiocarbohydrazide on MDA-MB-231 cells, and the results are shown in Figures S7 and S8 (Supporting Information). The IC_50_ value for cisplatin in MDA-MB-231 cells was calculated as 16.44 μM, which is quite higher than the IC_50_ value (0.41 μM) of complex **1** in the same cell line. This indicates the high cytotoxic potential of complex **1** compared to cisplatin. It can be clearly seen from the MTT assay performed with the free ligand (Figure S8, Supporting Information) that the cell viability of the TNBC cells is much higher as compared to its copper complex (**1**) as already shown in Figure 5B. In addition, cytotoxic activity of complex **1** was also investigated in a nonmalignant breast epithelial cell line MCF-10A and the results are shown in Figure S9 of the Supporting Information. The compound was found to be remarkably nontoxic in MCF-10A cells even at very high concentrations (75 μM) showing its selectivity toward TNBC (MDA-MB-231) cells. Overall, it can be concluded that the synthesized Cu­(II) complex **1** has high cytotoxic potential in MDA-MB-231 cells which substantiates further studies in this cell line.

**Figure 5:**
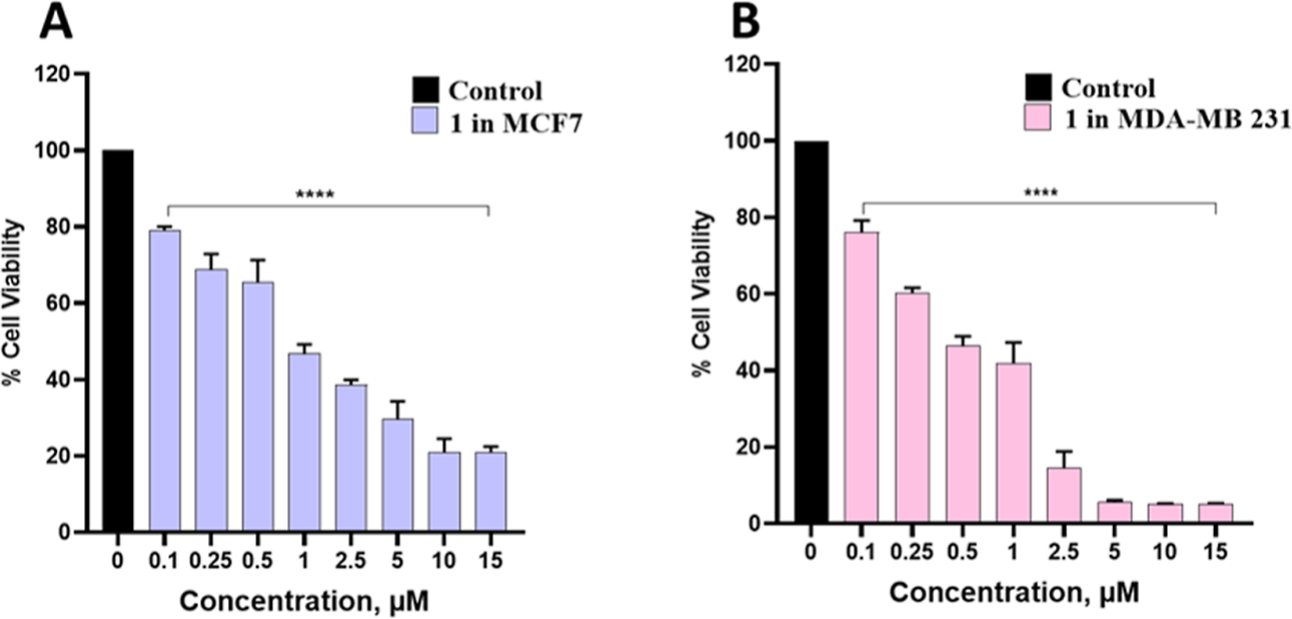
Percentage cell viability: (A) MCF-7 and (B) MDA-MB-231 vs concentrations of complex **1**. The statistical analysis was performed using GraphPad Prism. **** indicates *p* < 0.0001.

#### 2.7.2 Nuclear Staining

There was substantial damage to plasmid DNA as well as cancer cell death by treatment of complex **1** as seen from the gel electrophoresis assay and cell viability assay (vide supra). In order to confirm nuclear damage and understand the mode of cell death in cancer cells, the nucleus was stained with Hoechst-33342. This is a membrane-permeable nuclear dye that evenly stains the nucleus of cells and fluoresces blue.^55^ However, healthy cells do not produce much fluorescence upon staining with Hoechst, while apoptotic cells with chromosomal condensation and fragmentation results into effective staining and high intensity fluorescence thus differentiating between healthy and apoptotic cells.^57^

In Figure 6 effective staining was observed in cells treated with **1**, while healthy control cells took up much less stain. Further, there was a clear decline in cell density with an increase in the concentration of complex **1**. Arrows in Figure 6 point toward densely stained condensed chromatin and small fragments of nucleus which are all clear signs of apoptosis.

**Figure 6:**
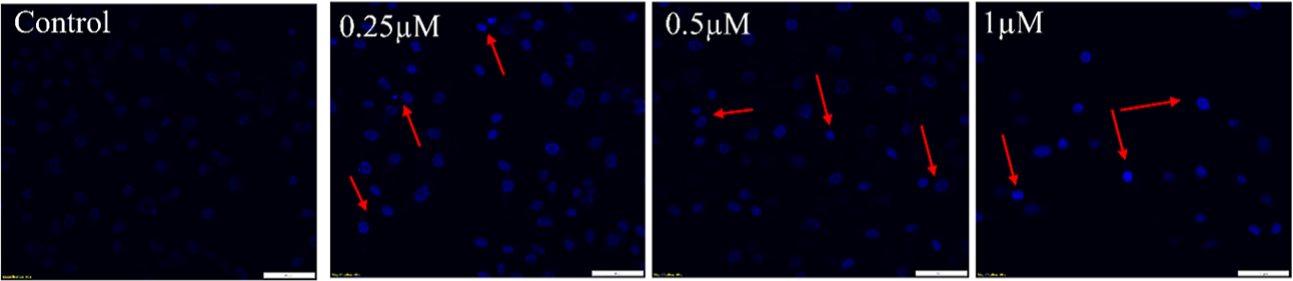
Changes in nuclear morphology of TNBC cells (MDA-MB-231) after treatment of complex **1** and observed under a confocal microscope with Hoechst 33342 stain. Scale bar of 50 μm.

#### 2.7.3 TUNEL Assay

This assay is a commonly used technique to detect DNA fragmentation, a hallmark of apoptosis.^58^ In order to confirm nuclear fragmentation, we performed DNA fragmentation using TUNEL-DAPI costaining. The TUNEL assay relies on labeling the exposed 3′–OH fragmented ends of DNA with tagged nucleotides using the enzyme TdT. This labeled DNA was visualized through fluorescence microscopy.^59^

In Figure 7, the green fluorescence due to TUNEL-labeled fragmented DNA increases with the increase in the concentration of complex **1**. The appearance of most TUNEL-positive cells in 0.25 μM suggests high DNA fragmentation at this concentration, which could lead to apoptosis.

**Figure 7:**
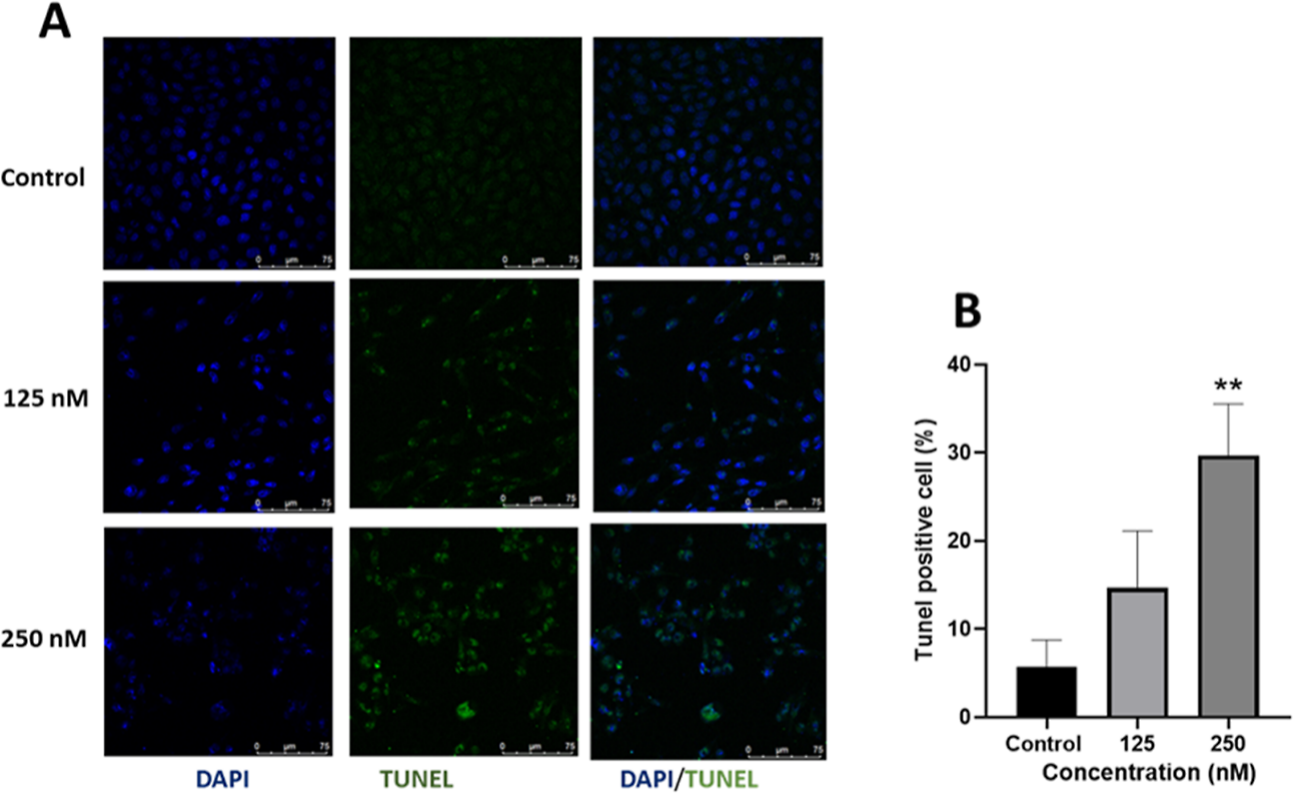
Evaluation of DNA fragmentation induced by complex **1** using the TUNEL assay: (A) DAPI staining, TUNEL staining, and merge image of untreated and treated MDA-MB-231 cells with complex **1**. (B) The graph shows amount of TUNEL-positive cells in response with increasing concentrations of **1**. Data are presented with mean ± SD (**) *p* < 0.001.

#### 2.7.4 PI Uptake

Propidium iodide (PI) is a molecule that can bind to DNA and emit fluorescence, but it does not have the ability to pass through cells that have an intact plasma membrane. This allows differentiation of live and dead cells.^60^ Hence, analyzing PI uptake gives an estimate of apoptotic cells with compromised cell membrane. In Figure 8, PI uptake by tested TNBC (MDA-MB-231) cells after 24 h exposure of complex **1** can be seen increasing in a dose-dependent manner. The PI uptake in cells that are predominantly close to apoptosis increased significantly in both treatment groups.

**Figure 8:**
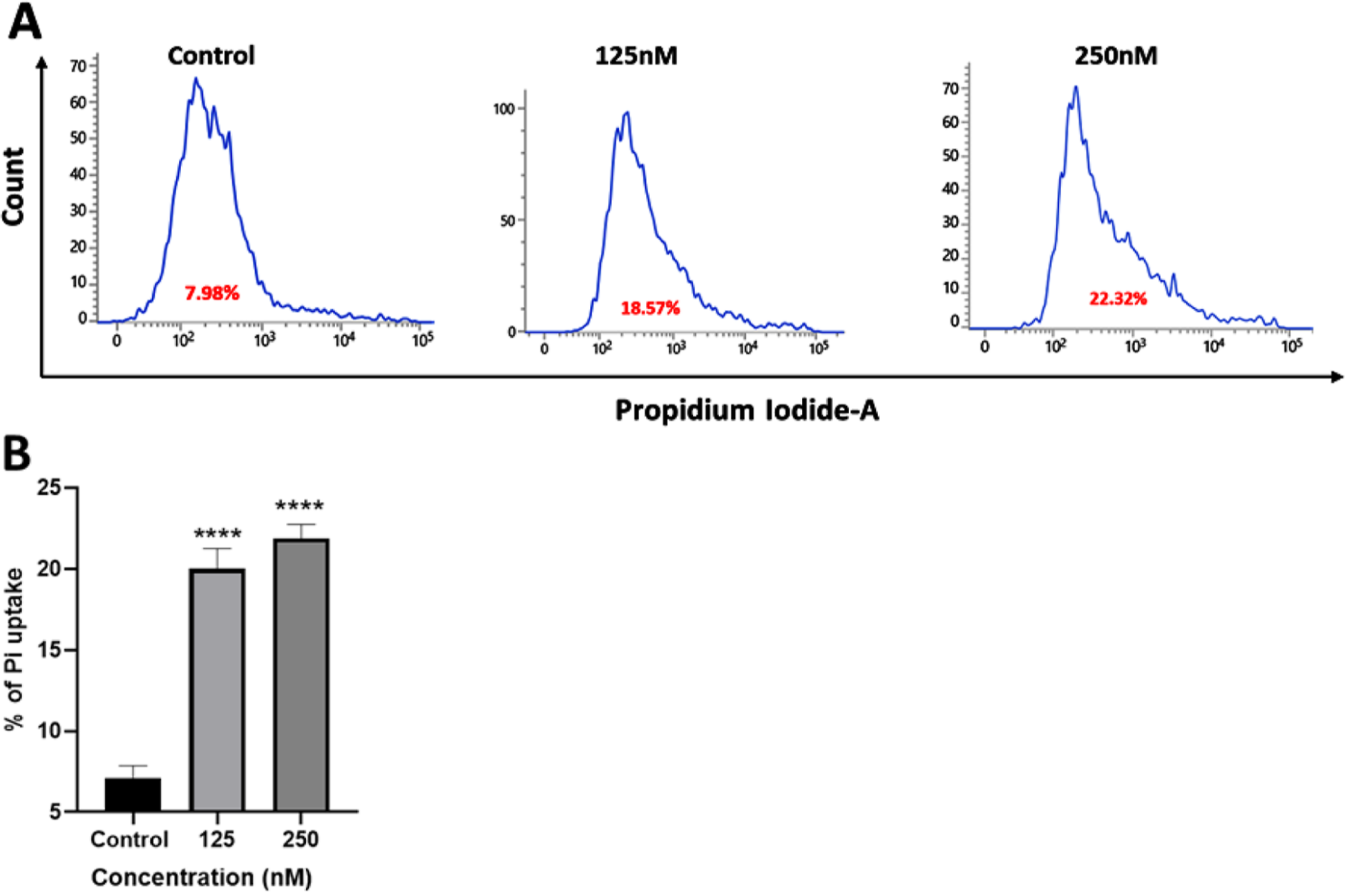
PI uptake by TNBC (MDA-MB-231) treated with **1 (K38)** for 24 h. (A) Flow cytometer analysis. (B) Bar diagram with percentage PI uptake vs the concentration of complex **1**. GraphPad Prism was used for analysis, where, **** represents *p* < 0.0001 indicating a high level of statistical significance.

#### 2.7.5 ROS Activity

It is reported that copper complexes may cause tumor cell apoptosis due to oxidative stress mediated by reactive oxygen species (ROS).^61,62^ In order to elucidate the relationship between cytotoxicity observed during the MTT assay and intracellular ROS activity, MDA-MB-231 cells were exposed to complex **1** and stained with DCF-DA. This is a nonfluorescent intracellular ROS indicator. In the presence of reactive oxygen species, DCF-DA gets oxidized to a cell-permeable fluorogenic probe DCF which has a green fluorescence, such that the intensity of this green fluorescence is correlated with intracellular ROS generation.^63–65^ Fluorescence microscopy was performed with DCF-DA stained MDA-MB-231 breast cancer cells.

In Figure 9 untreated control cells, *tert*-butyl hydrogen peroxide (TBHP) treated as the positive control, and 0.5 μM of complex **1**-treated cells are shown on DCF-DA staining. It can be observed that there is little or limited fluorescence in control cells compared to both the positive control and complex **1**-treated cells. Further, complex **1**-treated cells are rounded and show eminent green fluorescence similar to the positive control. This confirms that complex **1** has potential to generate ROS which may lead to oxidative stress, resulting in apoptosis.

**Figure 9:**
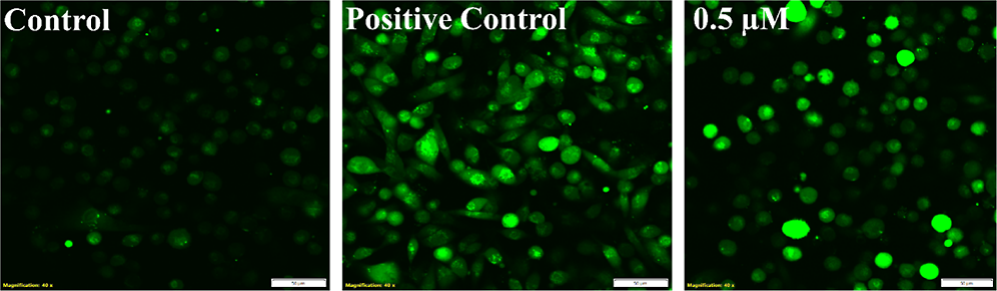
ROS activity determined by DCF-DA staining: fluorescence images showing untreated cells, positive control (TBHP), and 0.5 μM complex **1** treated cell line. Scale bar 50 μm.

#### 2.7.6 Cell Cycle Arrest

The cell cycle consists of G_0_, G1, S, G2, and M phase, where G_0_ is the quiescent stage of cell cycle. During G1, the cells increase in size. The S phase is the stage for DNA replication, G2 is the preparatory phase, and the actual cell division or mitosis takes place in the M phase.^66^ Upon DNA damage, cells undergo cell cycle arrest to repair damaged DNA^67,68^ Until then, cell cycle progression is stopped by p53.^69^ We have confirmed DNA damage with the help of gel electrophoresis, Hoechst staining, and the TUNEL assay. In order to understand the effects of the DNA damage on cell cycle progression, we conducted flow cytometry using fluorescence-activated cell sorting (FACS).

The effect of complex **1** on MDA-MB-231 cells can be seen in Figure 10 where the cell population in both S and G2/M phases is increasing in a dose-dependent manner. This clearly indicated that the DNA damages caused by complex **1** has led to cell cycle arrest in the S phase where DNA replication takes place, thus inhibiting DNA synthesis^70^ and the cells which escaped arrest from this phase got arrested in the next phase of the cell cycle which is the G2/M phase and could not undergo mitosis. Hence, the S and G2/M phase arrest has inhibited DNA replication, thus indicating that the proliferation of these breast cancer cells was restricted by activity of complex **1**.

**Figure 10:**
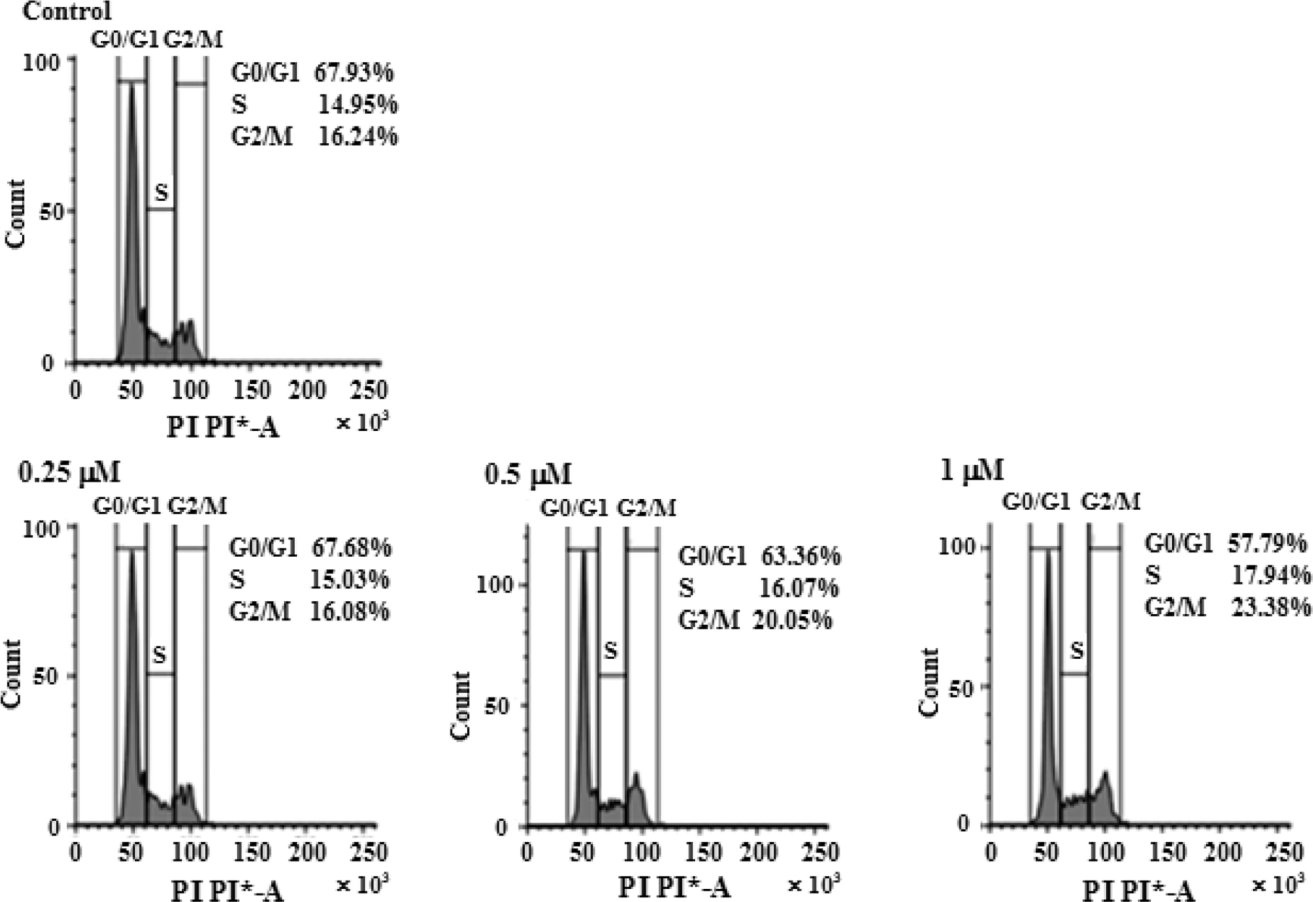
Effect of complex **1** treatment of 24 h on progression of the cell cycle as observed in TNBC cells (MDA-MB-231) through flow cytometric analysis.

#### 2.7.7 Western Blot Analysis

Apoptosis can progress through the caspase-dependent pathway as well as the independent pathway.^71^ To understand the role of caspases and the mechanism of cell death induced by copper complex **1,** we performed western blot analysis and examined the expression of some key markers of DNA damage and apoptosis like gamma H2AX, p53, PARP, and caspases.

The results of western blot are shown in Figure 11 where there is marked increase in the expression of phosphorylated gamma H2AX. This Phosphorylation is known to occur upon initiation of DNA fragmentation.^72,73^ Further, compared to the control, a clear sharp increase in p53 lane expression can be observed, confirming activation of p53 in response to cellular DNA damage and cell cycle arrest (Figure 11) by complex **1**. Further, mitochondrial cytochrome c release was accompanied by dose-dependent increased expression of caspase 9 and caspase 3 proves that cytochrome c is released from mitochondria to the cytosol, which led to caspase 9 activation and finally caspase 3. Apart from this, Figure 11 also shows the appearance of cleaved PARP fragments upon treatment with complex **1**. The 89 kDa fragment shows that cleavage of full length 116 kDa PARP1 has taken place, which is considered as a hallmark of caspase activation and apoptosis.^74^ These results further confirmed the apoptotic potential of complex **1** and strongly point toward the involvement of the caspase-mediated intrinsic apoptotic pathway that is primarily associated with the mitochondria.^75^

**Figure 11:**
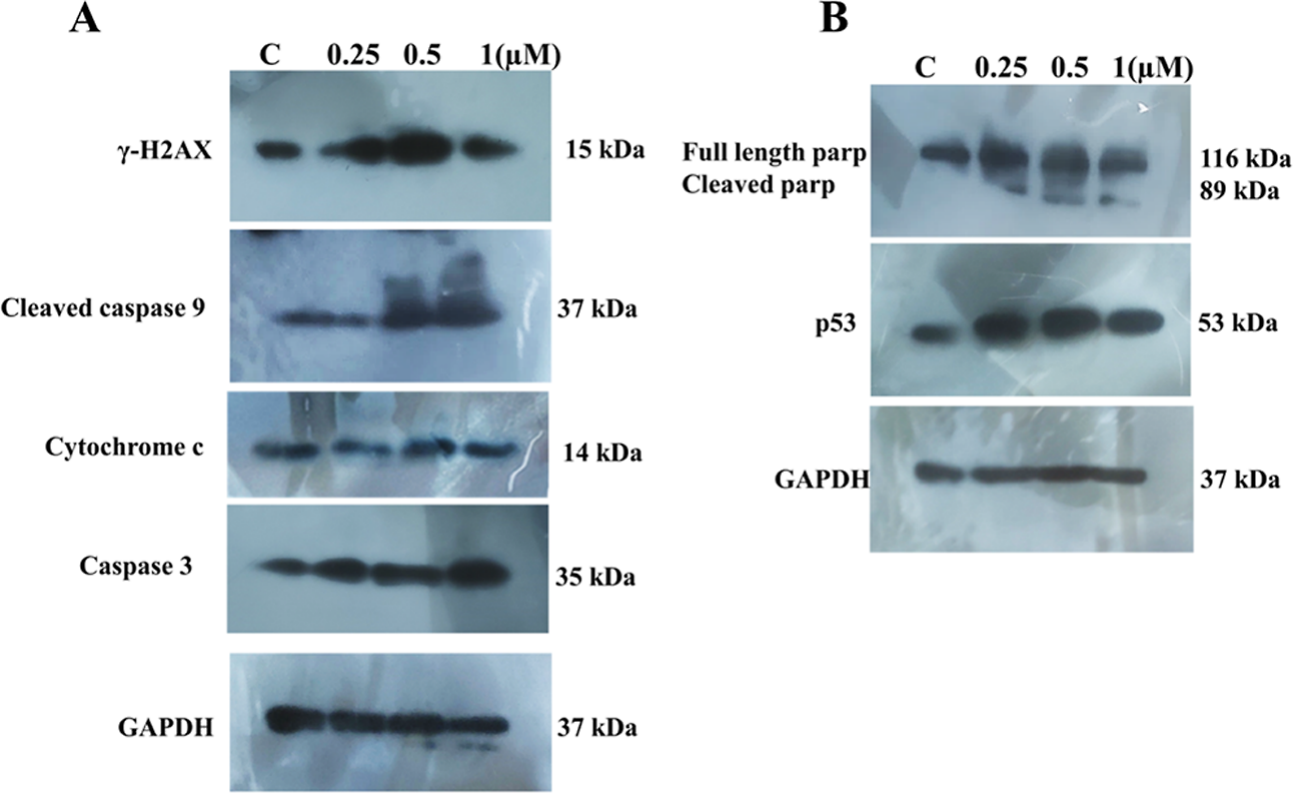
Analysis of protein expression using western blot for complex **1**-treated MDA-MB-231 cells: (A) showing the expression of Phosphorylated Gamma-H2AX, cleaved caspase 9, cytochrome c, caspase 3, and cleaved caspase 8 with GAPDH used as the loading control. (B) Shows PARP cleavage, P53 expression with GAPDH as the control.

#### 2.7.8 MitoTracker Assay

The intrinsic pathway of apoptosis relies mainly on the mitochondria to initiate the apoptotic cascade, and this process involves changes in mitochondrial mass.^76^ The MitoTracker assay can be used to track and measure changes in mitochondrial mass by flow cytometry. MitoTracker Green (MTG) was used in the present study, which is a cationic dye that selectively accumulates in the mitochondrial matrix by passing the plasma membrane of the mitochondria of live cells. Once inside the mitochondria, the chloromethyl groups of the MTG react with free thiol groups of cysteine residues on mitochondrial proteins, forming thioethers. This reaction causes the dye to covalently bind to mitochondrial protein, which results in fluorescence. The fluorescence intensity represents the mitochondrial mass.^77^

To study the changes in mitochondrial mass MDA-MB-231 cells were treated with complex **1** and post treatment, live cells were labeled with Invitrogen’s MitoTracker Green and fluorescence was acquired and analyzed using flow cytometry. Quantification of MitoTracker Green represents the total mass of the mitochondria present, which was observed to be less in complex-treated cells compared to the control (Figure 12). The data suggest that there could have been mitochondrial dysfunction or damage upon treatment with complex **1** which led to a decrease in total mitochondrial mass.

**Figure 12:**
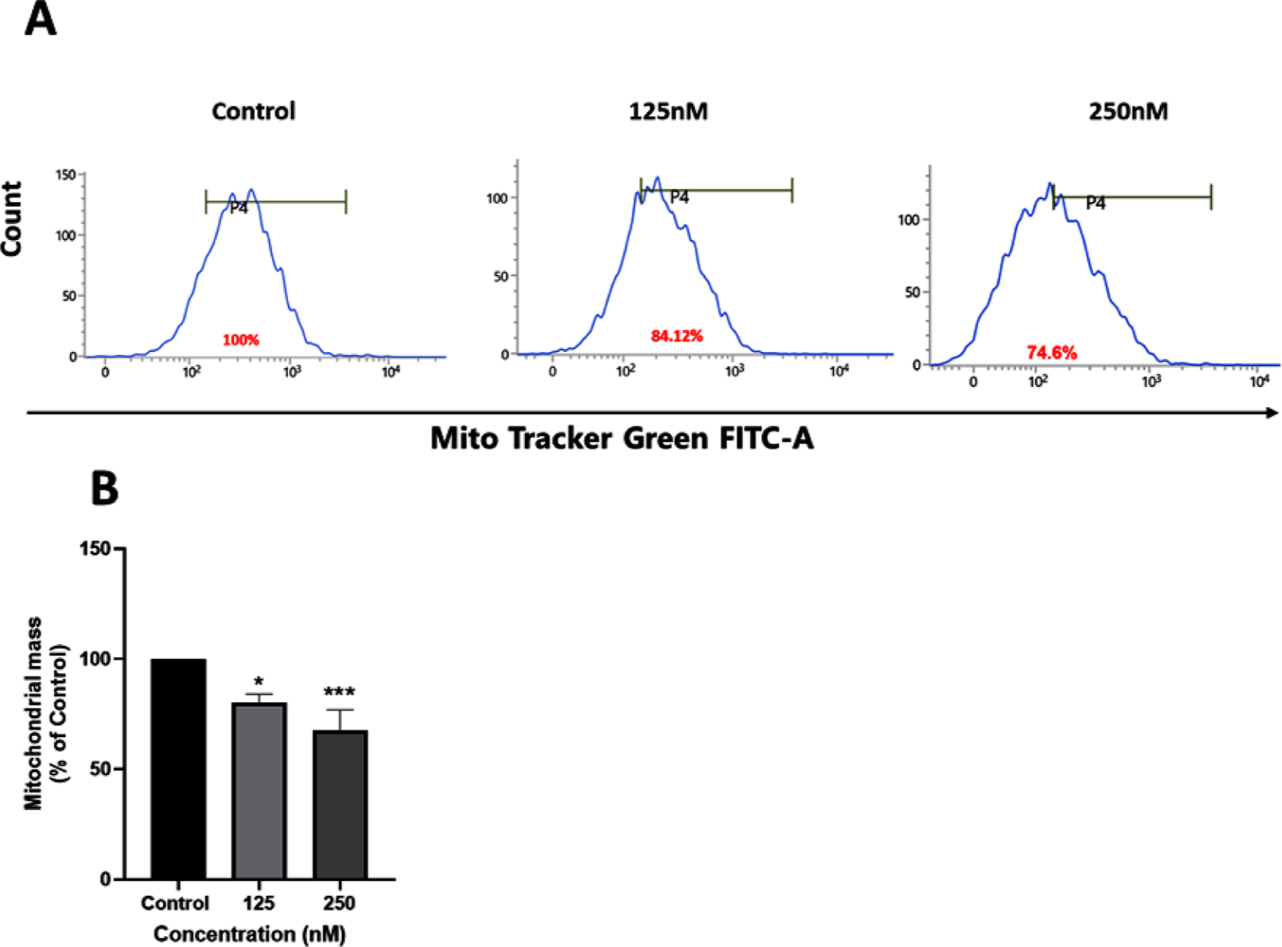
Analysis of mitochondrial mass using flow cytometry: (A) mitochondrial mass detected by the MitoTracker Green staining of control and complex **1-**treated MDA-MB-231 cells and (B) the intensity of MitoTracker Green fluorescence quantified to measure mitochondrial mass. Statistical analysis was performed with GraphPad Prism. Where, * indicates *p* < 0.05 and *** Mean value of represented groups are significantly decreased from the control (*p* < 0.001).

#### 2.7.9 MitoSox Assay

Mitochondria is an important source of intracellular reactive oxygen species (ROS).^78^ MitoSOX targets mitochondria and specifically detects superoxide, an important ROS in mitochondria,^77^ so it was used to study the effects of complex **1** on mitochondrial superoxide production by fluorescence microscopy as well as flow cytometry. In the present study, complex **1**-treated MDA-MB-231 cells were subsequently stained with Invitrogen MitoSOX green and counterstained with DAPI. The green fluorescence was produced as a result of oxidation of MitoSOX by superoxide, which was visualized under a confocal microscope. The intensity of the green fluorescence is proportional to the level of superoxide production in the mitochondria.

As seen in Figure 13, the green fluorescence intensity of MitoSOX has significantly increased with treatment of an increasing concentration of complex **K38** on MDA-MB-231 cells. This increase in MitoSOX-derived green fluorescence may be equated to mitochondrial superoxide (O_2_^•–^).

**Figure 13:**
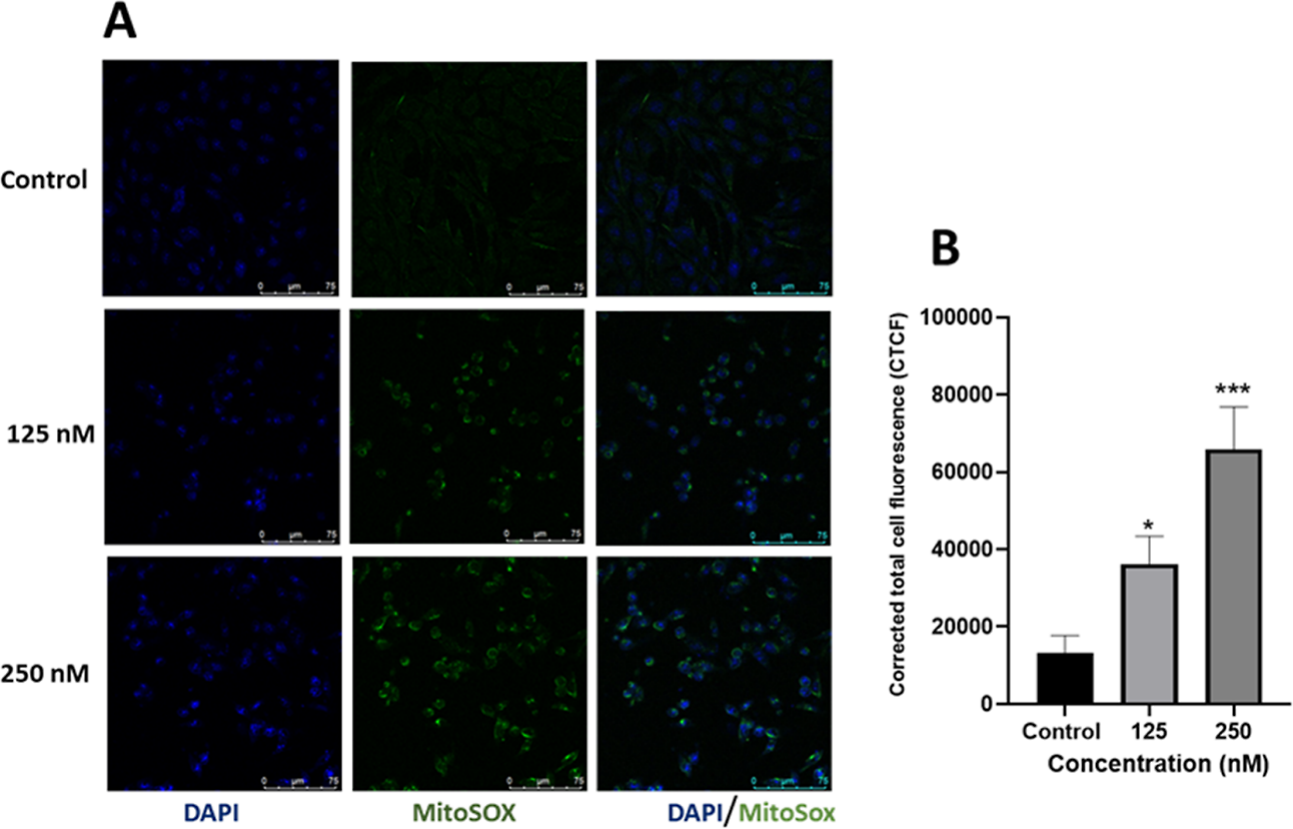
Mitochondrial superoxide levels in TNBC cells (MDA-MB-231) following exposure to complex **1 (K38)** by fluorescence microscopy: (A) **K38-**treated MDA-MB-231 cells stained with MitoSOX green and counterstained with DAPI. (B) Corrected total cell fluorescence (CTCF) at different doses of complex **1**. GraphPad Prism was used for statistical analysis, where, * represents *p* < 0.05 and *** for *p* < 0.001.

Flow cytometry allows the sensitive and reliable quantification of mitochondrial ROS (mROS). Consequently, flow cytometry was used to analyze the production of mROS simultaneously. MDA-MB-231 cells were treated with complex **1**, stained with live cell-permeant reagent MitoSOX red. Once inside the mitochondria, a constituent of MitoSOX, dihydroethidium is oxidized by superoxide forming 2-hydroxyethidium which fluoresces, and this fluorescence was acquired using flow cytometry. The flow cytometry results showing increase in mROS in complex **1-**treated cells (Figure 14) are in line with the observations made using fluorescence microscopy.

**Figure 14:**
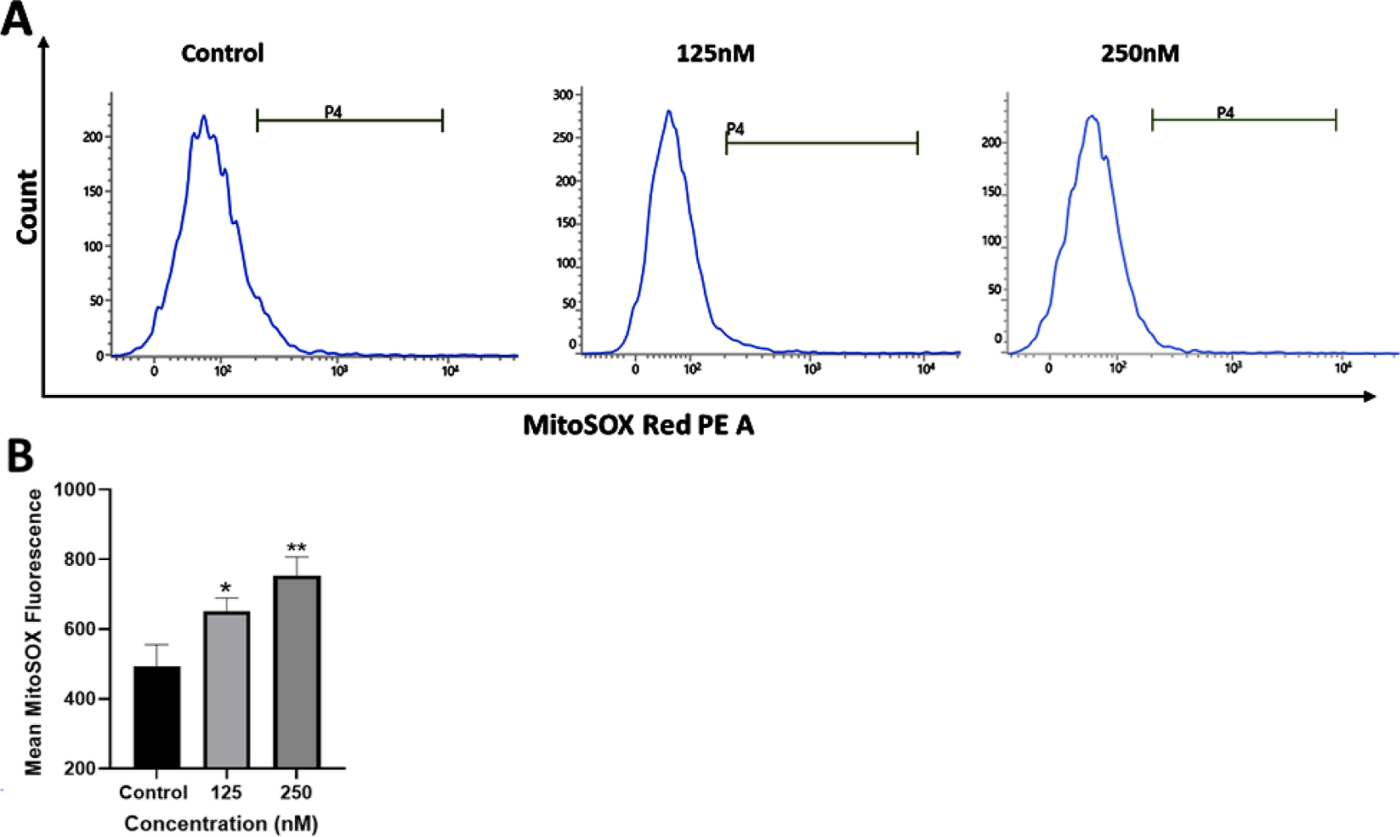
Analysis of mitochondrial superoxide production using flow cytometry: (A) MitoSOX based flow cytometric detection of mitochondrial ROS in control and MDA-MB-231 cells treated with complex **1 (K38)**. (B) Bar plot showing quantification of mitochondrial ROS levels. Statistical analysis performed with GraphPad Prism, where, * represents *p* < 0.05, ** represents *p* < 0.01.

These observations suggest that mitochondria can be considered as a source of complex **1**-induced ROS generation in the tested cancer cells. Thus, from our findings collectively we may say that in addition to DNA damage (vide supra), the inclusion of complex **1** may induce damage to mitochondria. Since high ROS levels can trigger apoptosis, treating cancer cells with such ROS-stimulating agents may prove to be a valuable addition to cancer-specific therapy.

#### 2.7.10 TMRM Assay

Mitochondrial attack is associated with drop in mitochondrial membrane potential (Δψ_m_).^76^ Therefore, Δψ_m_ is an important parameter to access mitochondrial function. To ascertain the ability of complex **1** to promote apoptosis in cancerous cells by a mitochondrial pathway, changes in the mitochondrial potential of MDA-MB-231 cells prior and after treatment with **1** were monitored using TMRM staining. TMRM (tetramethylrhodamine methyl ester) is a cell-permeant fluorescent dye that accumulates and fluoresces orange in polarized mitochondria. Fluorescence drops significantly on depolarization in apoptotic or metabolically stressed cells.

As seen in Figure 15, treatment of MDA-MB-231 cells with complex **1** resulted in significant decrease of TMRM fluorescence, which implies higher mitochondrial depolarization on treatment and resulting in subsequent reduction in mitochondrial membrane potential. Membrane depolarization and mitochondrial ROS (reactive oxygen species) production are related; excessive ROS synthesis can cause the mitochondrial membrane to collapse, which may result in cell malfunction or death.

**Figure 15:**
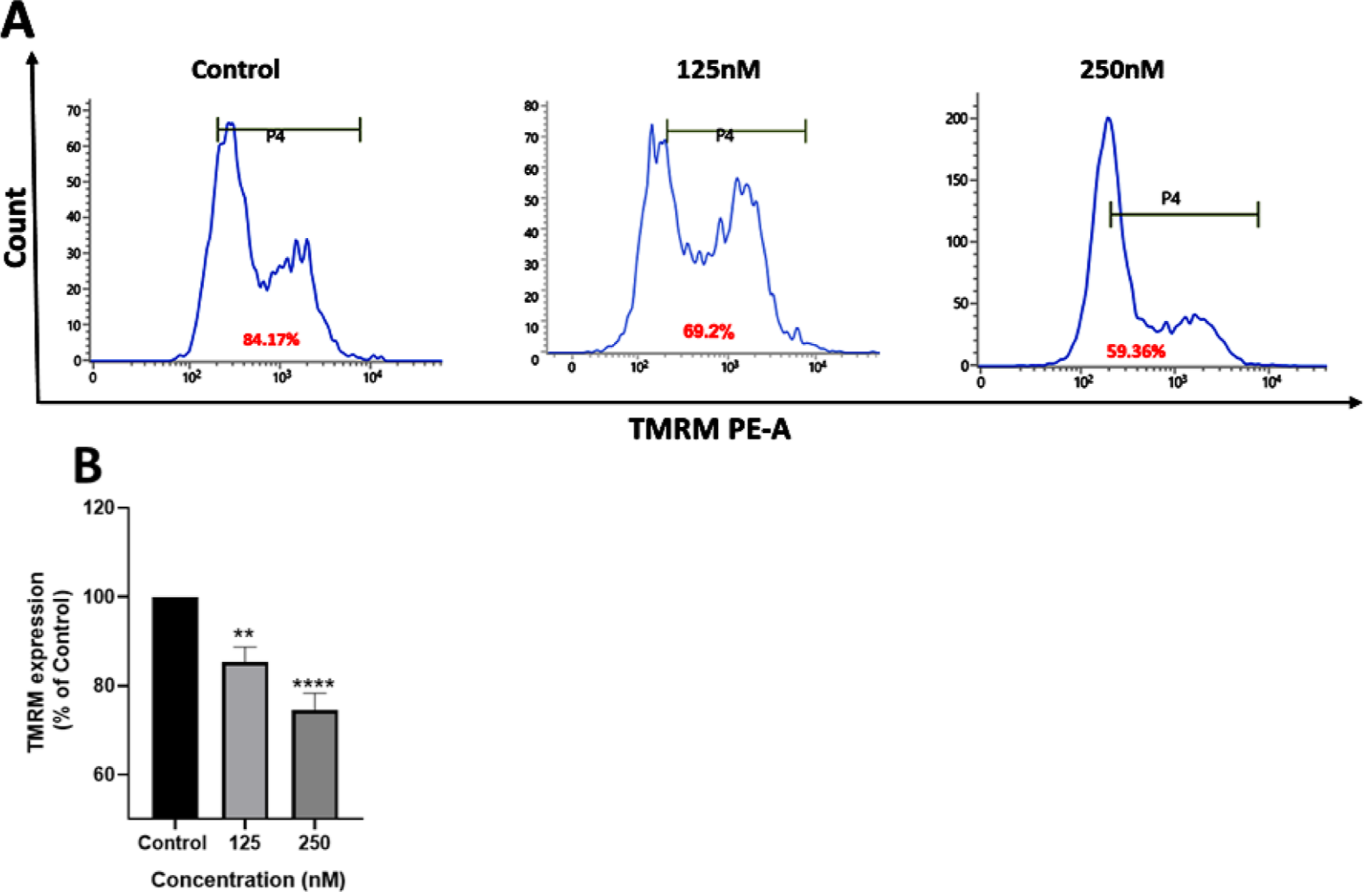
TMRM assay. (A) The graphs showing the change of TMRM fluorescence upon treatment of MDA-MB-231 cells with complex **1**, measured by FACS analysis. (B) Bar diagram shows decrease in TMRM expression with respect to the control. Statistical analysis was performed by GraphPad Prism, where, ** represents *p* < 0.01 and **** represents *p* < 0.0001 indicating a high level of statistical significance.

## 3 Conclusions

The synthesized Cu­(II) complex **1** was tested for DNA binding and cleavage abilities. The complex showed exceptionally good DNA binding potential wherein the intrinsic binding constant was of the same order as the classical intercalator EB. In addition, the complex showed good nuclease activity. Further, cytotoxicity studies revealed that this complex was highly cytotoxic to both MCF-7 and MDA-MB-231 breast cancer cell lines, exhibiting IC_50_ values in the submicromolar range, while this compound was found to be nontoxic in the nonmalignant breast epithelial MCF-10A cells showing its selectivity toward breast cancer cell lines. Hoechst and TUNEL imaging on MDA-MB-231 cells showed condensation, apoptotic bodies, and DNA fragmentation. In addition, ROS generation by complex **1** was also identified by DCFDA images. The mechanistic pathway involved in cell death studied through western blot analysis revealed that complex **1** led to apoptosis through the caspase cascade activation by the intrinsic mitochondrial pathway. Finally, complex **1** promoted mitochondrial ROS (mROS) production which disrupted redox homeostasis and decreased the mitochondrial mass. This imbalance triggered intrinsic apoptosis, highlighting the critical role of ROS in mitochondrial metabolism and cell death.

## 4 Materials and Methods

### 4.1 General Procedures

The compound was characterized by high-resolution mass spectroscopy (HRMS) using a Xevo-G2 XS QTOF mass spectrometer, Waters UHPLC system, and a Thermo Scientific Q Exactive hybrid quadrupole-Orbitrap mass spectrometer as well as by electron paramagnetic resonance (EPR) spectroscopy with the help of a JEOL, Japan Model: JES - FA200 ESR spectrometer with X and Q-band. Room-temperature solution EPR spectra were recorded using an aqueous cell, and frozen glass spectra were recorded in liquid nitrogen using a quartz dewar. Elemental analysis (C, H, and N) was conducted using a PerkinElmer model 2400 series II CHNS/O analyzer. The infrared (IR) spectrum was obtained with a Shimadzu IR Affinity-1 FT-IR spectrometer and electronic spectra were recorded on a Jasco V-570 UV/vis/NIR spectrophotometer using a pair of matched quartz cell of a path length of 1 cm. Fluorescence measurements were performed using a Jasco fluorescence spectrophotometer FP-8500. Conductivity was measured with the help of a Mettler Toledo dual conductivity/pH meter model SevenMulti equipped with Inlab 730 and Inlab 413 electrodes.

### 4.2 Materials

CT-DNA and cisplatin were acquired from Sigma. The plasmid pUC19 DNA was sourced from Thermo Fisher Scientific. The tested cell lines like MCF-7 and MDA-MB-231 were obtained from (NCCS), Pune, India. All antibodies used in western blot were purchased from Cell Signaling Technology. Remaining chemicals used in this study were of reagent grade.

### 4.3 Syntheses

#### 4.3.1 Synthesis of the Schiff Base 1,5-bis­(salicylidene)­thiocarbohydrazide (A), (*o*-HOC_6_H_4_CH=NNH)_2_C=S (H_3_L)

This Schiff base was prepared by modifying the method reported by Bustos et al.^27^ Salicylaldehyde (5 mmol) was added directly to a solution of thiocarbohydrazide (2.5 mmol) in 50 mL of absolute ethanol, and the reaction mixture was stirred at RT for 2 h. The pale yellow solid that separated was filtered through a G-3 sintered glass crucible, washed well with ethanol, and dried. This was recrystallized from methanol. Yield: 68%; mp 191 °C. Anal. Calcd (%) for C_15_H_14_N_4_O_2_S: C (57.30), H (4.49), N (17.83); found, (%): C (57.41), H (4.47), N (17.90).

#### 4.3.2 Synthesis of the Dinuclear Cu­(II) Complex [(*o*-phen)­Cu­(L)­Cu­(*o*-phen)]­(NO_3_) (1)

0.2415 g (0.001 mol) of Cu­(NO_3_)_2_·3H_2_O was reacted with 0.2 g (0.001 mol) of 1,10-phenanthroline monohydrate in 30 mL of methanol and this reaction mixture was slowly added to a solution of 0.157 g (0.0005 mol) of the Schiff base ligand 1,5-bis­(salicylidene)-thiocarbohydrazide in 50 mL of methanol at room temperature (RT) while the reaction mixture turned green. This reaction mixture was stirred at RT for 24 h, and the dark green solid that was separated from the solution was filtered through a G-4 crucible, washed with methanol, and dried. This dark green compound was then thoroughly washed with water, dried in air, then washed with methanol followed by acetonitrile, and dried under vacuum. This compound was then recrystallized from methanol at 4 °C. Yield: 65%. Elemental analysis: C_39_H_27_N_9_O_5_SCu_2_: calcd; (found), C 54.37 (54.97); H 3.16 (3.23); N 14.64 (14.82) %. ESI–MS in CH_3_OH (*m*/*z*): calcd for [Cu_2_ (HL)^3–^(CH_3_OH)_2_ + H]^+^ (504.31) corresponding to [Cu_2_ (C_15_H_11_N_4_O_2_S) (CH_3_OH)_2_ + H]^+^, observed at 504.09; calcd for [(Cu_2_((HL)^3–^ (sal)^−^ (CH_3_OH)_2_ + H]^+^ (624.43) corresponding to Cu_2_(C_15_H_11_N_4_O_2_S)­(C_7_H_5_O_2_) (CH_3_OH)_2_ + H]^+^, observed at 624.67; calcd for [(*o*-phen)­Cu_2_(H L′)^2–^ (NO_3_)^−^ (CH_3_OH)]^+^ (711.48) corresponding to [(C_12_H_8_N_2_) Cu_2_(C_15_H_10_N_4_O_2_S) (NO_3_)^−^ (CH_3_OH)]^+^, observed at 711.61; calcd for [(*o*-phen)­Cu_2_ (HL)^3–^(sal)^−^]^+^ (739.64) corresponding to [(C_12_H_8_N_2_) Cu_2_ (C_15_H_11_N_4_O_2_S) (C_7_H_5_O_2_)]^+^, observed at 739.65; calcd for [(*o*-phen)­Cu_2_(H L′)^2–^ (CH_3_OH)­(NO_3_)^−^ + K]^+^ (750.48) corresponding to [(C_12_H_8_N_2_) Cu_2_(C_15_H_10_N_4_O_2_S) (CH_3_OH)­(NO_3_)^−^ + K]^+^, observed at 750.44; calcd for [(*o*-phen)­Cu_2_(H_2_L)^2–^ (sal)^−^ (H_2_O) + H)]^+^ (759.64) corresponding to [(C_12_H_8_N_2_) Cu_2_ (C_15_H_12_N_4_O_2_S) (C_7_H_5_O_2_) (H_2_O) + H]^+^, observed at 760.12; calcd for [(*o*-phen)­Cu_2_ (H L′)^2–^ (sal)^−^ (H_2_O)+ K]^+^ (795.60) corresponding to [(C_12_H_8_N_2_) Cu_2_(C_15_H_10_N_4_O_2_S) (C_7_H_5_O_2_) (H_2_O)+ K]^+^, observed at 795.53; calcd for [(*o*-phen)_2_ Cu_2_(HL)^3–^(CH_3_OH)_2_ + H]^+^ (863.73) corresponding to [(C_12_H_8_N_2_)_2_ Cu_2_ (C_15_H_11_N_4_O_2_S) (CH_3_OH)_2_ + H]^+^, observed at 864.13; calcd for [(*o*-phen)_2_ Cu_2_(HL)^3–^ (TSC)]^+^ (889.82) corresponding to [(C_12_H_8_N_2_)_2_ Cu_2_ (C_15_H_11_N_4_O_2_S) (CH_5_N_3_S)]^+^, observed at 889.49; calcd for [(*o*-phen)_2_Cu_2_(HL)^3–^ (NO_3_)^−^ (H_2_O)_3_ + H]^+^ (915.69) corresponding to [M+3H_2_O + H]^+^ or [(C_12_H_8_N_2_)_2_ Cu_2_ (C_15_H_11_N_4_O_2_S) (NO_3_)^−^ (H_2_O)_3_ + H]^+^, observed at 915.98.

### 4.4 DNA Binding

In order to analyze the DNA binding ability of the synthesized metal complex **1**, a Jasco V-570 UV/vis/NIR spectrophotometer was used. The stock for calf thymus DNA (CT-DNA) was prepared by overnight soaking it in Tris–HCl buffer at pH 7.4. The purity of this DNA was confirmed by an absorbance ratio (*A*_260_/*A*_280_) of 1.86, indicating that it was free of protein contamination. The CT-DNA concentration was determined by dividing the UV absorbance at 260 nm by the molar extinction coefficient ε_260_ = 6600 L mol^–1^ cm^–1^. For the electronic absorbance titration experiment, increasing amounts of CT-DNA (concentration range: 0 to 25 μM) were added to a fixed concentration of complex **1** (1 × 10^–5^ M) which was dissolved in DMSO (1%) diluted in 10 mM Tris–HCl. The binding constant (*K*_b_) was then calculated using the Wolfe–Shimmer equation.

### 4.5 Viscosity Measurement

DNA binding of complex **1** and cisplatin was studied by measurement of the viscosity using an Ostwald viscometer. Flow time was measured for each sample thrice. The samples consisted of 100 μM CT DNA dissolved in Tris–HCl with increasing concentrations (0–100 μM) of complex **1** dissolved in DMSO (1%) and diluted in Tris–HCl, pH 7.4. The calculation for viscosity was done using the formula η = (*t* – *t*_0_)/*t*_0_, here *t*_0_ is the flow time of buffer alone and *t* is the flow time of each sample. A plot of (η/η_0_)^1/3^ against [complex]/[DNA] was drawn, where η is the viscosity of DNA with the complex and η_0_ is the viscosity of DNA without the complex.

### 4.6 EB Displacement Assay

This technique is frequently used to identify the intercalation ability of a compound. EB reacts specifically with DNA, emitting strong fluorescence which may be quenched using a competitive molecule such as a metal complex. A Jasco spectrofluorometer FP-8500 was used to understand EB displacement by complex **1**. An incremental amount of complex **1** dissolved in DMSO (0.5%) diluted in 10 mM Tris–HCl with concentrations of 0, 1, 2, 3, 4, and 5 μM was added to a solution of 2 μM EB and 50 μM CT DNA. Keeping 2 min as incubation time, the fluorescence spectra were measured at an excitation wavelength λ_ex_ = 510 nm.

### 4.7 Nuclease Activity

Gel electrophoresis was used to investigate the ability of complex **1** to cleave pUC19 DNA. The pUC19 DNA was prepared in a Tris–HCl buffer with 50 mM NaCl at pH 7.43. To study DNA cleavage through the oxidative pathway, 200 ng of pUC19 DNA was treated with varying concentrations of **1** (ranging from 0.25 to 1 μM) and 2 μL of H_2_O_2_. The complex **1** was dissolved in DMF (0.5%) diluted in 50 mM Tris–HCl (pH 8). The reaction mixtures were then incubated at 37 °C for 2 h. To study the hydrolytic DNA-cleaving ability without external agents or light, 200 ng of pUC19 DNA was directly treated with increasing concentrations of **1** (ranging from 0 to 120 μM) and incubated at 37 °C for 3 h. Electrophoresis for the samples was performed in TAE buffer for 1.5 h at 60 V. Following this, the gel stained with 0.5 μg/mL EB was photographed under UV light.

### 4.8 MTT Assay

In order to perform cytotoxicity analysis, MDA-MB-231 cells were grown in DMEM with 10% FBS and incubated in an incubator at 37 °C with 5% CO_2_ until they reach approximately 80% confluency. Once the cells were confluent, 2 × 10^4^ cells were seeded in 96-well plates in triplicates and left to attach overnight in the incubator. Complex **1** was dissolved in DMSO (0.2%) and then diluted with DMEM. The concentrations of complex **1** used in the MTT assay were as follows: 0.1, 0.25, 0.5, 1, 2.5, 5, 10, and 15 μM. The cytotoxicity assay was also conducted with cisplatin and the free Schiff base 1,5-bis­(salicylidene)­thiocarbohydrazide. These were dissolved in DMSO (0.2%) and then diluted with DMEM. The concentrations used for cisplatin and the free ligand were 0, 1.5, 3, 6, 12, 25, 50, and 100 μM. These concentrations were used in the designated wells ensuring same final volume in each well. The cytotoxic activity of complex **1** using the MTT assay was also investigated in the nontumorigenic MCF-10A cell line, in order to determine its selectivity. These cells were cultured in a humidified incubator with 5% CO_2_ at 37 °C in DMEM/F12 media supplemented with 5% horse serum, epidermal growth factor (20 ng/mL), insulin (10 μg/mL), hydrocortisone (0.5 mg/mL), cholera toxin (100 ng/mL), and penicillin/streptomycin. Briefly, 3000 cells were seeded per well in 96-well plates (Nunc) and incubated at 37 °C with various concentrations of complex **1**. After 24 h treatment, fresh MTT reagent was prepared by dissolving it in PBS (5 mg/mL) and a working stock of 0.5 mg/mL was added to each well for 4 h. After this, MTT was removed, and formazan crystals were dissolved in DMSO. Absorbance reading was taken for each well by a microplate reader at 570 nm.

### 4.9 Nuclear Staining

Fluorescent images of the nucleus of MDA-MB-231 cells were captured using a Hoechst 33342. Cells were plated at a density of 5 × 10^5^ cells in 35 mm confocal dishes. Upon attachment/overnight incubation, the cells were replenished with fresh media, and the cells were treated with a specified concentration of compound **1** (**K38**) for 24 h. After the incubation period, the cells were washed with 1X PBS, fixed with 4% paraformaldehyde, and permeabilized with 0.1% triton X-100. Then, the cells were stained with Hoechst-33342 (1 μg/mL) in the dark for 30 min. Followed by 1X PBS rinse thrice, the fluorescence microscope (OLYMPUS CKX53) was used to observe the cells in the confocal dishes.

### 4.10 TUNEL Assay

The TUNEL assay was performed according to a previously standardized protocol with slight modifications.^59^ Briefly, the cells (MDA-MB-231) were treated with complex **1** for 24 h. Subsequently, the cells were washed with PBS, fixed with 4% formaldehyde, and equilibrated for 5–10 min. Then, the cells were labeled with TdT reaction mix (Qiagen) for 60 min at 37 °C. Subsequently, the experiment was terminated using stop solution, counterstained with DAPI, and mounted on glass slides using mounting media (Sigma). Slides were visualized using confocal microscopy (Leica Microsystems, Wetzlar, Germany) with excitation and emission at 490 and 520 nm, respectively.

### 4.11 Propidium Iodide Uptake

This assay was conducted with reference to a previously standardized protocol.^59^ Cells were seeded and then, upon attachment, treated with complex **1** for 24 h. After this, cells were suspended in PBS and incubated with PI (2 μg/mL) in the dark at 37 °C for 20 min. The stained cells were analyzed by a flow cytometer (BD FACSLyric, San Jose, CA, USA).

### 4.12 Intracellular ROS

TNBC cells (MDA-MB-231) were plated at a density of 5 × 10^5^ in a 35 mm confocal dish. Upon attachment/overnight incubation, the cells were replenished with fresh media, and the cells were treated with a specified concentration of compound **1** (**K38**) and 25 μM *t*-BHP (*tert*-butyl hydrogen peroxide) for 6 h. After the incubation period, the cells were washed with 1X PBS and stained with 10 μM of DCFDA and left inside an incubator in the dark for 30 min. It was rinsed with 1X PBS thrice and then images were taken using a confocal microscope (OLYMPUS CKX53).

### 4.13 Cell Cycle Arrest

The cell cycle analysis was performed using FACS. 1 × 10^6^ cells were plated in a 6-well plate and left to attach overnight in a humidified CO_2_ incubator. Next day, medium was replaced and wells were supplied with complex **1** treatment. After 24 h of incubation, cells were harvested with the help of trypsin and a mechanical cell scraper. The obtained cell pellet was subjected to 70% chilled ethanol treatment followed by centrifugation. Next, 10 μg of RNase was incubated in the resuspended pellet at 37 °C for 0.5 h. Finally, the cells were stained with propidium iodide for 30 min in the dark and analyzed by BD FACS MELODY.

### 4.14 Western Blot Analysis

The first step for western blot analysis was protein extraction from complex **1**treated cells using RIPA lysis buffer and 1X protease inhibitor. The protein concentration was measured using the Bradford reagent. Next, gel electrophoresis of an equal concentration of this cell lysate was carried out and the proteins from agarose gel were transferred to a PVDF membrane. Finally, the blot was incubated in indicated primary antibody (1:1000) overnight at 4 °C and secondary antibody (1:3000) for 2 h at room temperature. In the end, the blots were developed on an X-ray film in the dark room.

### 4.15 MitoTracker Assay

The MitoTracker assay was performed according to previously standardized protocol.^77^ The cells (MDA-MB-231) were treated with complex **1** for 24 h. Post treatment, live cells were labeled with Invitrogen’s MitoTracker Green (100 nM) for 20 min in phenol-free media at 37 °C. Subsequently, cells were trypsinized, washed, and resuspended in PBS. MitoTracker Green fluorescence was acquired using flow cytometry (BD FACSLyric, San Jose, CA, USA) with excitation and emission at 490 and 516 nm, respectively.

### 4.16 MitoSOX Assay Was Done by Flow Cytometry and Microscopy

#### 4.16.1 Flow Cytometry

The MitoSOX assay was carried out according to a previously standardized protocol with slight modification.^77^ The MDA-MB-231 cells were treated with complex **1** for 24 h. Subsequently, the cells were trypsinized and centrifuged at 800 rpm for 5 min. Then, cells were stained with Invitrogen’s MitoSOX red (1 μM) for 20 min at 37 °C. Finally, the cells were washed and resuspended in 300 μL of PBS. The MitoSOX red fluorescence was acquired using flow cytometry (BD FACSLyric, San Jose, CA, USA) with excitation and emission at 396 and 610 nm, respectively.

#### 4.16.2 Microscopy

The MDA-MB-231 cells were treated with complex **1** for 24 h. Subsequently, the live cells were stained with the Invitrogen MitoSOX Green (1 μM) working concentration in phenol red free media from 5 mM stock for 20 min at 37 °C. After incubation, cells were washed with PBS. Finally, the cells were washed and counterstained with DAPI (1 μg/mL) and mounted on glass slides using immunofluorescence mounting media (MP Biomedicals, Santa-Ana, CA, USA). Finally, the slides were visualized under a confocal microscope (Leica Microsystems, Wetzlar, Germany) with excitation and emission at 488 and 510 nm, respectively, and the images were captured.

### 4.17 MitoProbe TMRM Assay

This assay was performed according to a previously standardized protocol.^77^ MDA-MB-231 cells were treated with complex **1** for 24 h. After the completion of treatment, cells were trypsinized and centrifuged at 800 rpm for 5 min. Then, cells are stained with MitoProbe TMRM (20 nM in phenol red free media) for 20 min at 37 °C. The cells were washed with PBS and resuspended in 300 μL of PBS and analyzed by flow cytometry (BD FACSLyric, San Jose, CA, USA) with an absorbance peak at 548 nm and an emission peak at 574 nm.

## Supplementary Material



## Notes

Characterization showing abbreviations used in HRMS spectra and species involved in HRMS spectra; HRMS spectra; IR spectra; electronic spectra; stability of **1** in Tris–HCl and DMEM; X-band EPR spectra; relative specific viscosity of DNA vs cisplatin; MTT assay of cisplatin in MDA-MB-231 cells; MTT assay of the free ligand in MDA-MB-231 cells; and MTT assay of **1** in nonmalignant breast epithelial cells MCF-10A (PDF)

## Abbreviations

DMF: *N*,*N*-dimethylformamide
DMSO: dimethyl sulfoxide
EB: ethidium bromide
MTT: 3-(4,5-dimethylthiazol-2-yl)-2,5-diphenyltetrazolium bromide
PI: propidium iodide
TUNEL: terminal deoxynucleotidyl transferase dUTP nick-end labeling
TdT: terminal deoxynucleotidyl transferase
DCFDA: 2’,7’-dichlorofluorescein diacetate
TMRM: tetra methyl-rhodamine methyl ester
