# Supplementary material for: Targeting Triple Negative Breast Cancer with a Dinuclear Copper(II) Thiocarbohydrazone Complex: Efficacy Evaluation and Cellular Response
Source: ACS Omega. 2025 Sep 3;10(36):41342–58. doi: 10.1021/acsomega.5c04277 (PMC12444681; doi:10.1021/acsomega.5c04277)
Supplement: Supplementary file 1 [file ao5c04277_si_001.pdf]

Supporting Information

Targeting triple negative breast cancer with a dinuclear copper(II) thiocarbohydrazone complex: efficacy evaluation and cellular response

Kumudini Paliwal[a], Abinash Swain[b], Durga Prasad Mishra[b*], Manjuri Kumar[a*]

[a]*Department of Chemical Engineering, Birla Institute of Technology and Science Pilani, K.K. Birla Goa Campus, Zuarinagar, Sancoale, Goa 403726, India*

[b]*Cell Death Research Laboratory, Endocrinology Division, CSIR-Central Drug Research Institute, B.S. 10/1, Sector-10, Jankipuram Extension, Lucknow, Uttar Pradesh 226031, India*

Scheme S1: **Following species¶ are involved or detected directly or indirectly in HRMS spectra**

$H_4L = C_{15}H_{14}N_4O_2S$, MW = 314.39	TCHz-sal (H_2L'') : $C_8H_{10}N_4OS$, MW = 210.27
$(H_3L)^{1-} = C_{15}H_{13}N_4O_2S$, MW = 313.39	$(HL'')^{1-} = C_8H_9N_4OS$, MW = 209.27
$(H_2L)^{2-} = C_{15}H_{12}N_4O_2S$, MW = 312.39	$(L'')^{2-} = C_8H_8N_4OS$, MW = 208.27
$(HL)^{3-} = C_{15}H_{11}N_4O_2S$, MW = 311.39	TCHz = CH_6N_4S, MW = 106.15
$(L)^{4-} = C_{15}H_{10}N_4O_2S$, MW = 310.39	TSC-sal = $C_8H_9N_3OS$, MW = 195.25
	$(\text{TSC-sal})^{2-} = C_8H_7N_3OS$, MW = 193.25
$H_3L' = C_{15}H_{12}N_4O_2S$, MW = 312.39	(sal) = $C_7H_6O_2$, MW = 122.12
$(H_2L')^{1-} = C_{15}H_{11}N_4O_2S$, MW = 311.39	$(\text{sal})^{1-} = C_7H_5O_2$, MW = 121.12
$(HL')^{2-} = C_{15}H_{10}N_4O_2S$, MW = 310.39	TSC = CH_5N_3S, MW = 91.13
o-phen = ($C_{12}H_8N_2$), MW = 180.21	Thiourea = CH_4N_2S MW = 76

¶Abbreviations used:

o-phen = 1,10-phenanthroline, TSC = thiosemicarbazide, TCHz = thiocarbohydrazide, sal = salicylaldehyde.

Scheme S2: Following species are involved or detected directly or indirectly in HRMS spectra

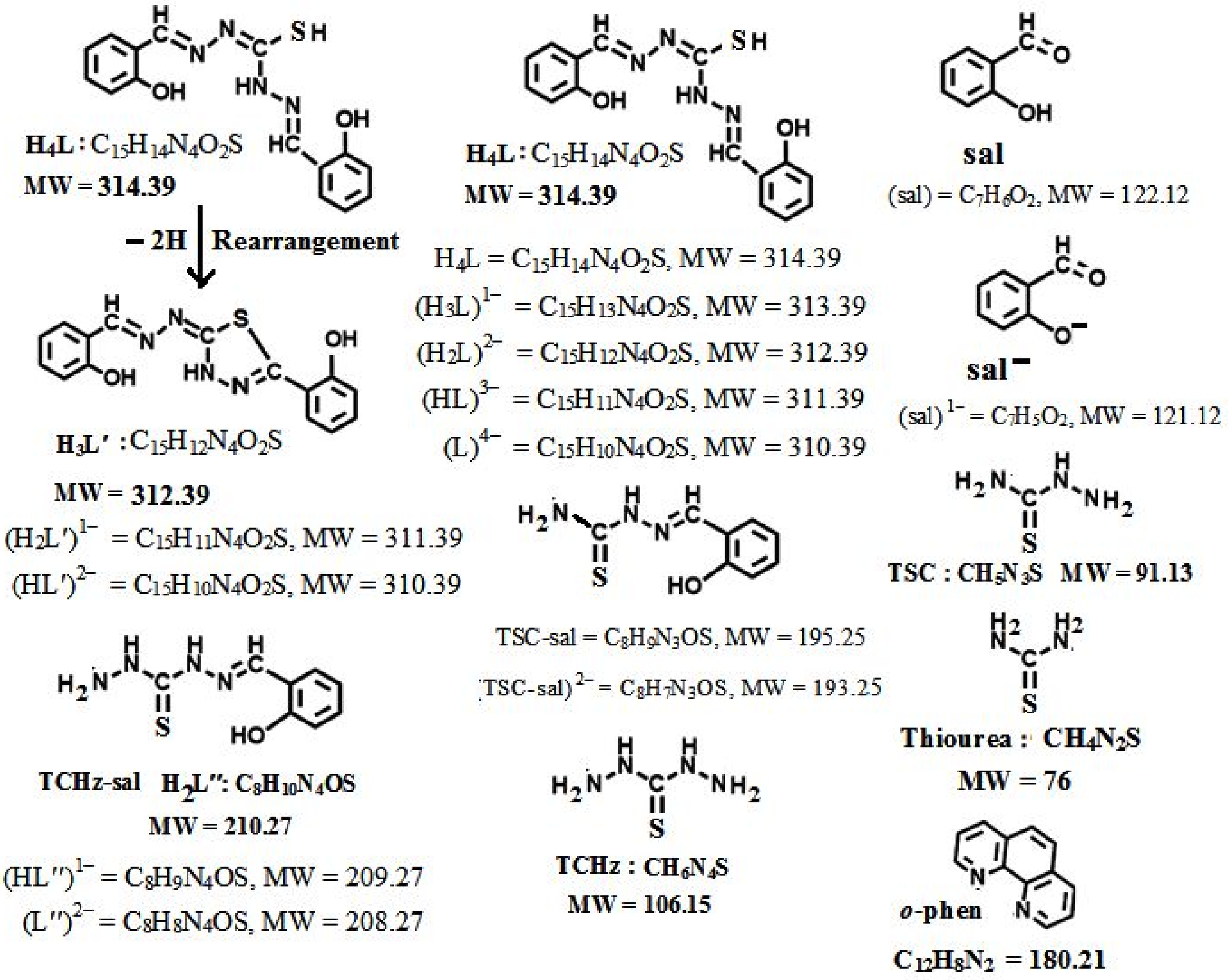

¶Abbreviations used:

o-phen = 1,10-phenanthroline, TSC = thiosemicarbazide, TCHz = thiocarbohydrazide, sal = salicylaldehyde.

A

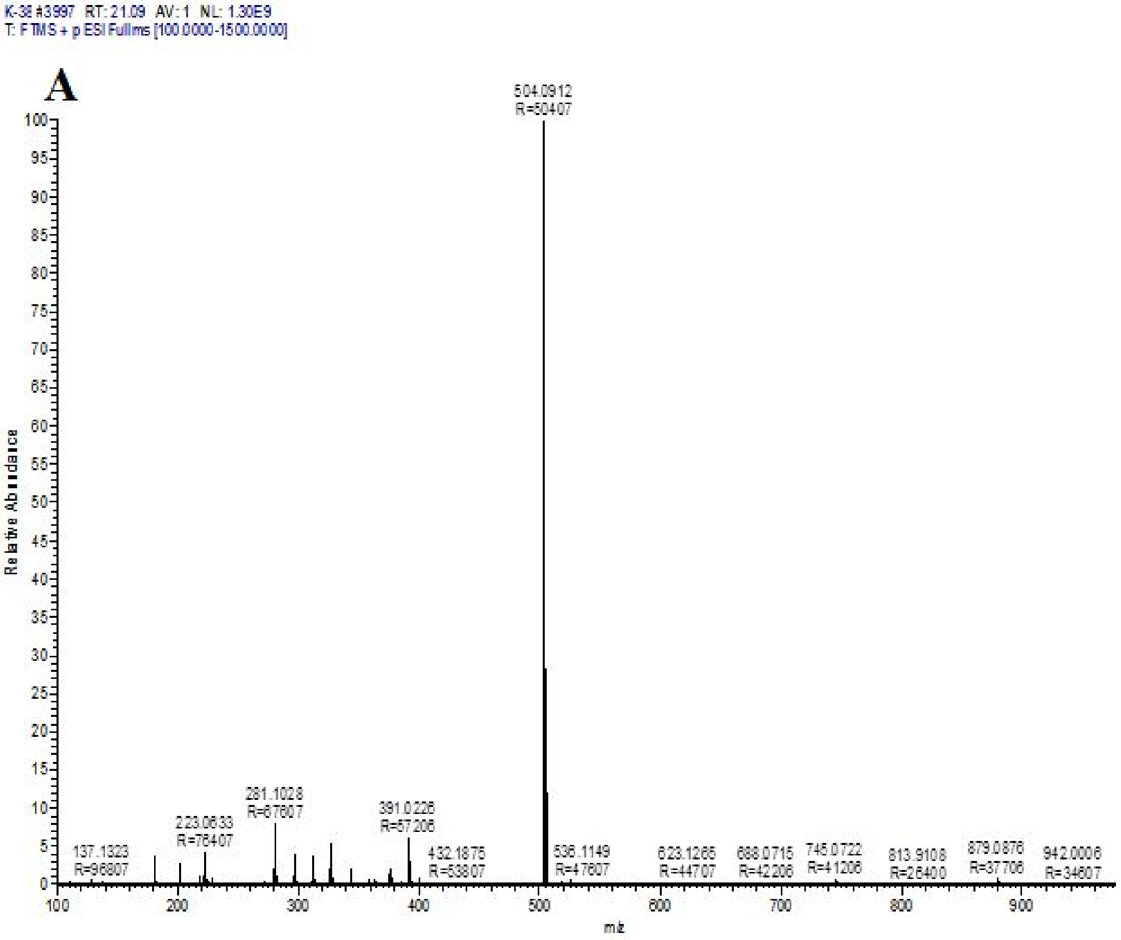
K-38 #3997 RT: 21.09 AV: 1 NL: 1.30E9
T: FTMS + p ESI Full ms [100.0000-1500.0000]
504.0912
R=50407
281.1028
R=67607
223.0633
R=76407
391.0226
R=57206
137.1323
R=96807
432.1875
R=53807
536.1149
R=47607
623.1265
R=44707
688.0715
R=42206
745.0722
R=41206
813.9108
R=26400
879.0876
R=37706
942.0006
R=34607
Relative Abundance
m/z

B

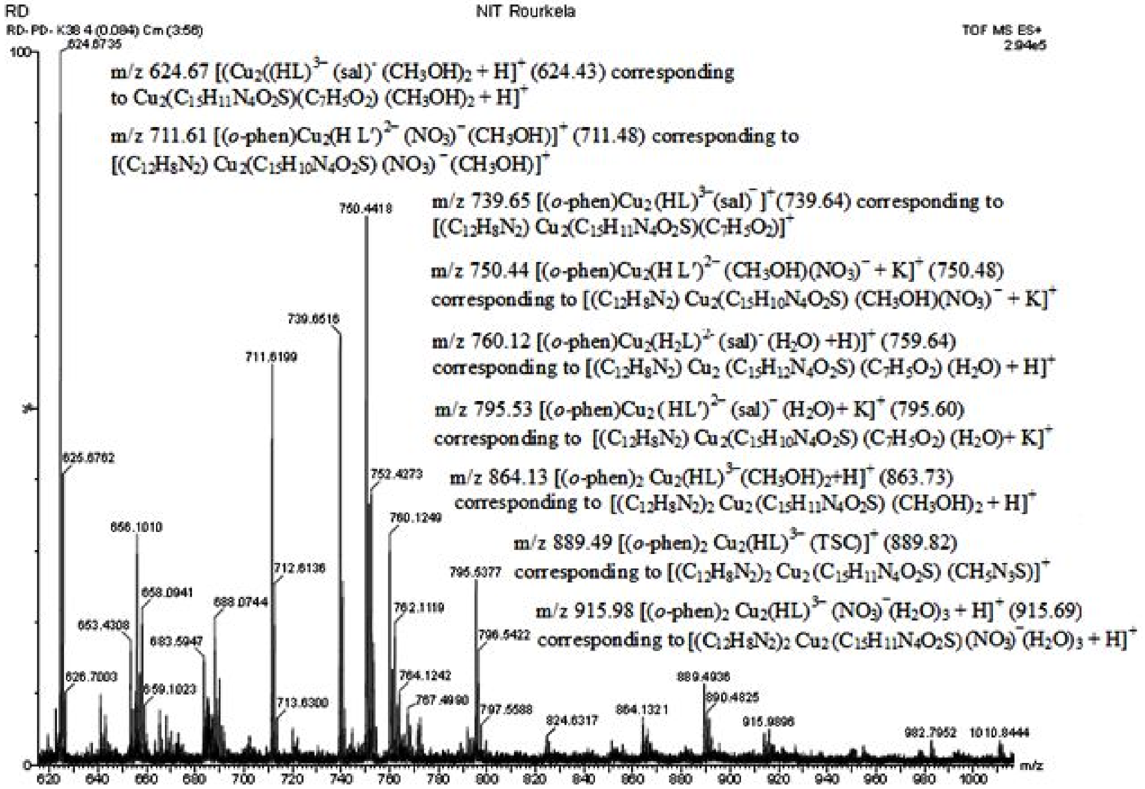
RD
NIT Rourkela
RD-PD-K38 4 (0.084) Cm (3:56)
TOF MS ES+
2.94e5
m/z 624.67 [(Cu$_2$((HL)$^{3-}$ (sal)$^-$ (CH$_3$OH)$_2$ + H]$^+$ (624.43) corresponding
to Cu$_2$(C$_{15}$H$_{11}$N$_4$O$_2$S)(C$_7$H$_5$O$_2$) (CH$_3$OH)$_2$ + H]$^+$
m/z 711.61 [(o-phen)Cu$_2$(H L')$^{2-}$ (NO$_3$)$^-$(CH$_3$OH)]$^+$ (711.48) corresponding to
[(C$_{12}$H$_8$N$_2$) Cu$_2$(C$_{15}$H$_{10}$N$_4$O$_2$S) (NO$_3$)$^-$(CH$_3$OH)]$^+$
m/z 739.65 [(o-phen)Cu$_2$(HL)$^{3-}$(sal)$^-$]$^+$(739.64) corresponding to
[(C$_{12}$H$_8$N$_2$) Cu$_2$(C$_{15}$H$_{11}$N$_4$O$_2$S)(C$_7$H$_5$O$_2$)]$^+$
m/z 750.44 [(o-phen)Cu$_2$(H L')$^{2-}$ (CH$_3$OH)(NO$_3$)$^-$ + K]$^+$ (750.48)
corresponding to [(C$_{12}$H$_8$N$_2$) Cu$_2$(C$_{15}$H$_{10}$N$_4$O$_2$S) (CH$_3$OH)(NO$_3$)$^-$ + K]$^+$
m/z 760.12 [(o-phen)Cu$_2$(H$_2$L)$^{2-}$ (sal)$^-$ (H$_2$O) +H)]$^+$ (759.64)
corresponding to [(C$_{12}$H$_8$N$_2$) Cu$_2$ (C$_{15}$H$_{12}$N$_4$O$_2$S) (C$_7$H$_5$O$_2$) (H$_2$O) + H]$^+$
m/z 795.53 [(o-phen)Cu$_2$ (HL')$^{2-}$ (sal)$^-$ (H$_2$O)+ K]$^+$ (795.60)
corresponding to [(C$_{12}$H$_8$N$_2$) Cu$_2$(C$_{15}$H$_{10}$N$_4$O$_2$S) (C$_7$H$_5$O$_2$) (H$_2$O)+ K]$^+$
m/z 864.13 [(o-phen)$_2$ Cu$_2$(HL)$^{3-}$(CH$_3$OH)$_2$+H]$^+$ (863.73)
corresponding to [(C$_{12}$H$_8$N$_2$)$_2$ Cu$_2$(C$_{15}$H$_{11}$N$_4$O$_2$S) (CH$_3$OH)$_2$ + H]$^+$
m/z 889.49 [(o-phen)$_2$ Cu$_2$(HL)$^{3-}$ (TSC)]$^+$ (889.82)
corresponding to [(C$_{12}$H$_8$N$_2$)$_2$ Cu$_2$ (C$_{15}$H$_{11}$N$_4$O$_2$S) (CH$_5$N$_3$S)]$^+$
m/z 915.98 [(o-phen)$_2$ Cu$_2$(HL)$^{3-}$ (NO$_3$)$^-$(H$_2$O)$_3$ + H]$^+$ (915.69)
corresponding to [(C$_{12}$H$_8$N$_2$)$_2$ Cu$_2$ (C$_{15}$H$_{11}$N$_4$O$_2$S) (NO$_3$)$^-$(H$_2$O)$_3$ + H]$^+$
624.6735
625.6762
626.7003
656.1010
658.0941
653.4308
659.1023
683.5947
688.0744
711.6199
712.6136
713.6300
739.6516
750.4418
752.4273
760.1249
762.1119
764.1242
767.4990
795.6377
796.5422
797.5588
824.6317
864.1321
889.4936
890.4825
915.9896
982.7952
1010.8444
m/z

Figure S1. HRMS spectra of **1** (**K38**) in methanol: (A) m/z 504.09 [Cu_2 $(HL)^{3-}(CH_3OH)_2$+H]$^+$ (504.31) corresponding to [Cu_2 ($C_{15}H_{11}N_4O_2S$) $(CH_3OH)_2$+H]$^+$. (B) m/z 624.67 [(Cu_2(($HL)^{3-}$ (sal)$^-$ $(CH_3OH)_2$ + H]$^+$ (624.43) corresponding to Cu_2($C_{15}H_{11}N_4O_2S$)($C_7H_5O_2$) $(CH_3OH)_2$ + H]$^+$; m/z 711.61 [(*o*-phen)Cu_2(H L′)$^{2-}$ $(NO_3)^-$ $(CH_3OH)]^+$ (711.48) corresponding to [($C_{12}H_8N_2$) Cu_2($C_{15}H_{10}N_4O_2S$) $(NO_3)^-$ $(CH_3OH)]^+$; m/z 739.65 [(*o*-phen)Cu_2 $(HL)^{3-}$(sal)$^-$]$^+$ (739.64) corresponding to [($C_{12}H_8N_2$) Cu_2 ($C_{15}H_{11}N_4O_2S$) ($C_7H_5O_2$)]$^+$; m/z 750.44 [(*o*-phen)Cu_2(H L′)$^{2-}$ (CH_3OH)$(NO_3)^-$ + K]$^+$ (750.48) corresponding to [($C_{12}H_8N_2$) Cu_2($C_{15}H_{10}N_4O_2S$) (CH_3OH)$(NO_3)^-$ + K]$^+$; m/z 760.12 [(*o*-phen)$Cu_2$$(H_2L)^{2-}$ (sal)$^-$ (H_2O) +H)]$^+$ (759.64) corresponding to [($C_{12}H_8N_2$) Cu_2 ($C_{15}H_{12}N_4O_2S$) ($C_7H_5O_2$) (H_2O) + H]$^+$; m/z 795.53 [(*o*-phen)Cu_2 (H L′)$^{2-}$ (sal)$^-$ (H_2O)+ K]$^+$ (795.60) corresponding to [($C_{12}H_8N_2$) Cu_2($C_{15}H_{10}N_4O_2S$) ($C_7H_5O_2$) (H_2O)+ K]$^+$; m/z 864.13 [(*o*-phen)$_2$ $Cu_2$$(HL)^{3-}$$(CH_3OH)_2$+H]$^+$ (863.73) corresponding to [$(C_{12}H_8N_2)_2$ Cu_2 ($C_{15}H_{11}N_4O_2S$) $(CH_3OH)_2$ + H]$^+$; m/z 889.49 [(*o*-phen)$_2$ $Cu_2$$(HL)^{3-}$ (TSC)]$^+$ (889.82) corresponding to [$(C_{12}H_8N_2)_2$ Cu_2 ($C_{15}H_{11}N_4O_2S$) (CH_5N_3S)]$^+$; m/z 915.98 [(*o*-phen)$_2$ $Cu_2$$(HL)^{3-}$ $(NO_3)^-$ $(H_2O)_3$ + H]$^+$ (915.69) corresponding to [M+3H_2O+H]$^+$ or [$(C_{12}H_8N_2)_2$ Cu_2 ($C_{15}H_{11}N_4O_2S$) $(NO_3)^-$ $(H_2O)_3$ + H]$^+$.

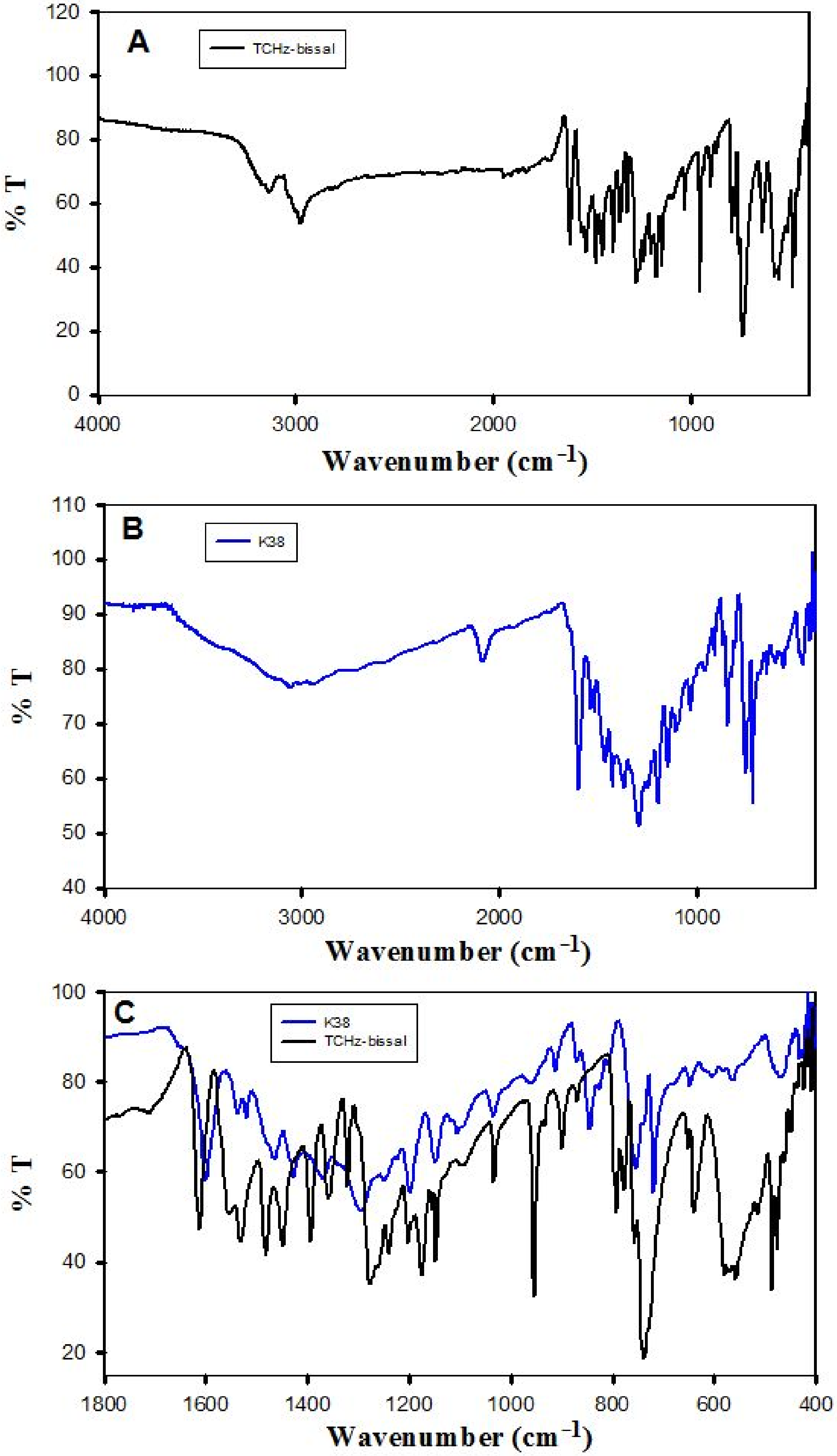

A
TCHz-bissal
% T
Wavenumber (cm^{-1})
B
K38
% T
Wavenumber (cm^{-1})
C
K38
TCHz-bissal
% T
Wavenumber (cm^{-1})

Figure S2. IR spectra: (A) Schiff base ligand 1,5-bis(salicylidene)thiocarbohydrazide. (B) Cu(II) complex **1** (**K38**). (C) Overlay of the Schiff base ligand and **1** (**K38**) in the 1800 to 400 cm^{-1} region.

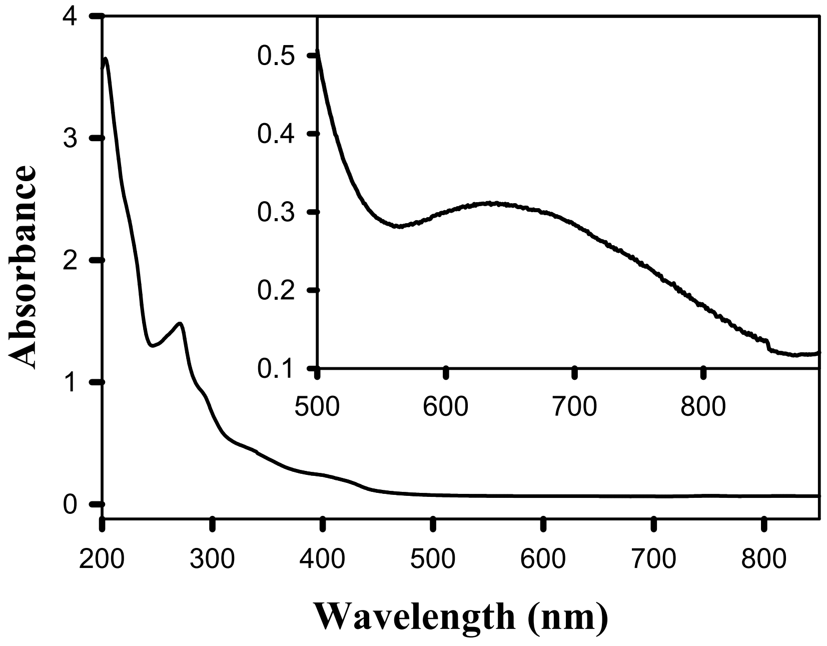

Figure S3. Electronic spectra of **1** in methanol. [**K38**] = 5 × 10^{-5} M, inset [**K38**] = 1×10^{-3} M.

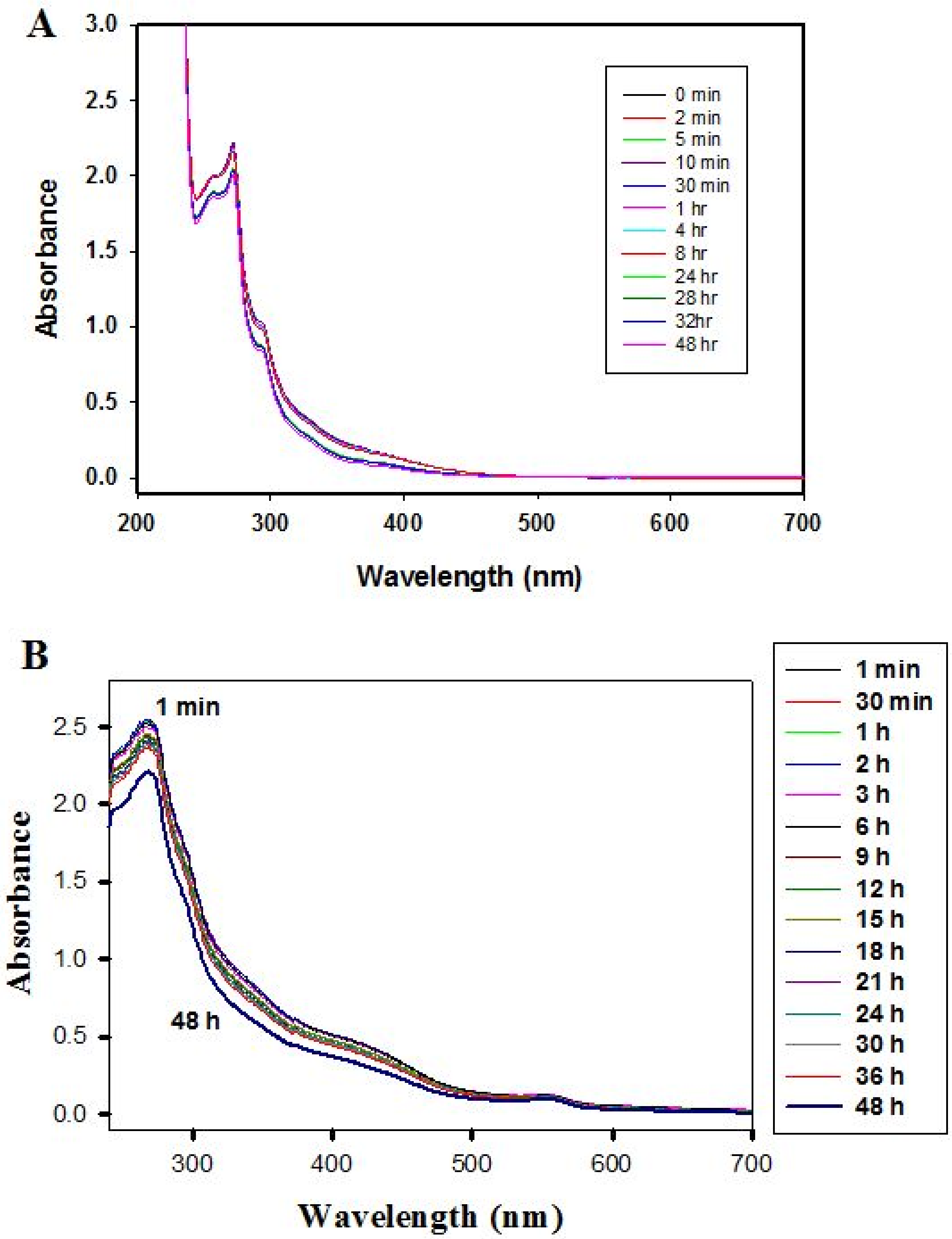

Figure S4. (A) Stability study in Tris-HCl pH 7.44, [**K38**] = 1×10^{-5} M. (B) Stability in DMEM for 48 h, [**K38**] = 1×10^{-5} M.

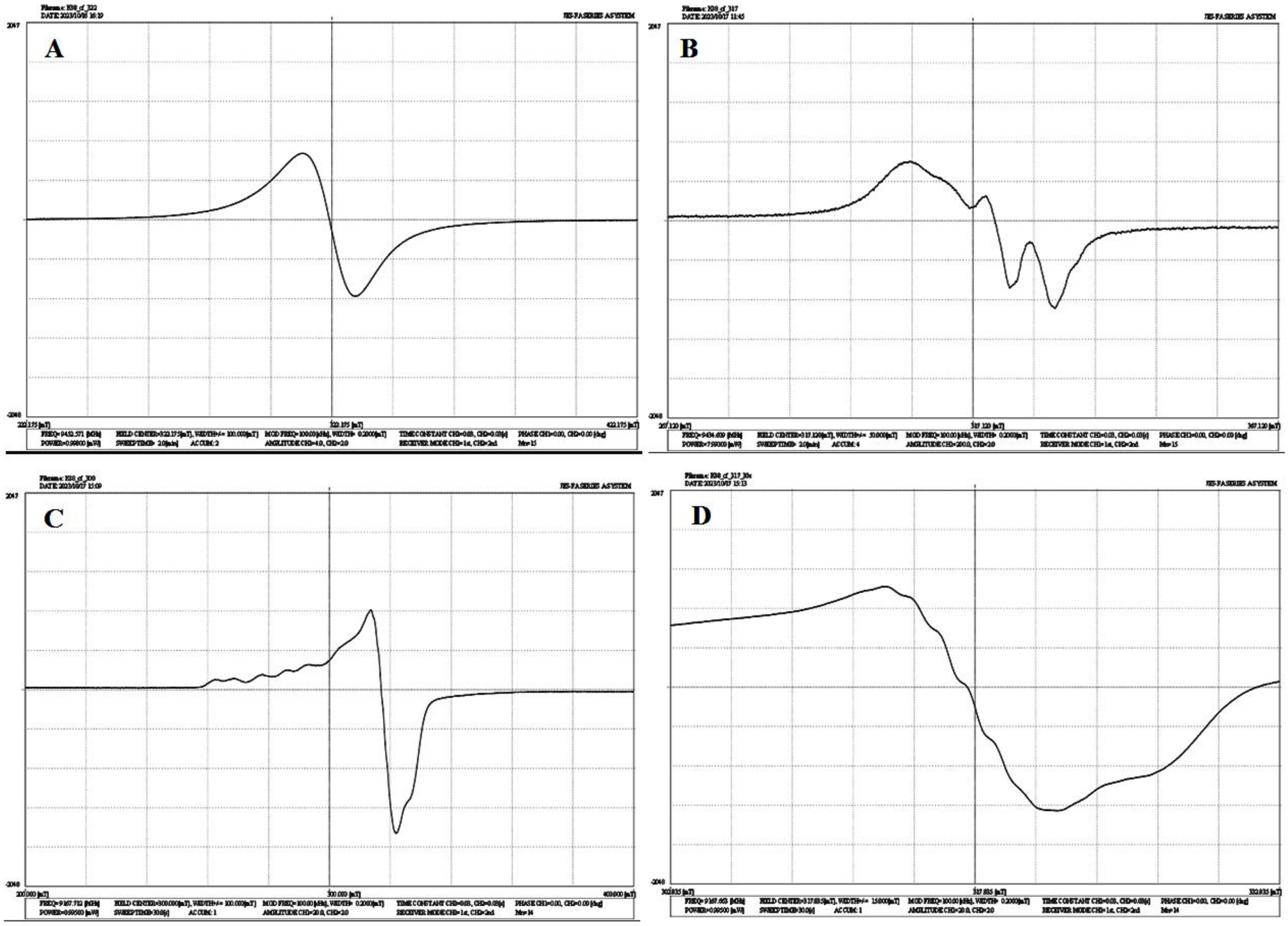

Figure S5. X-band EPR spectra of **1** (**K38**): (A**)** RT powder spectrum with all parameters. (B) Methanol solution spectrum at RT with all parameters. (C) Methanol frozen glass spectrum at LNT with all parameters. (D) Central line of the LNT spectrum shown in C is expanded to show the N-hyperfine lines.

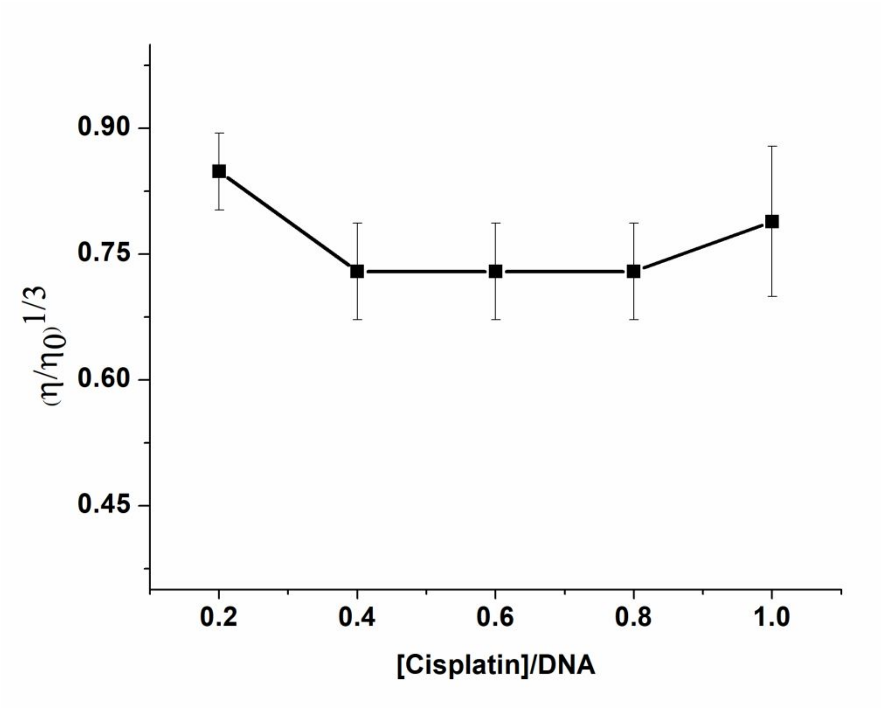

Figure S6. Relative specific viscosity $(\eta/\eta_0)^{1/3}$ vs. [cisplatin]/[DNA]. [CT DNA] = 100 μM..

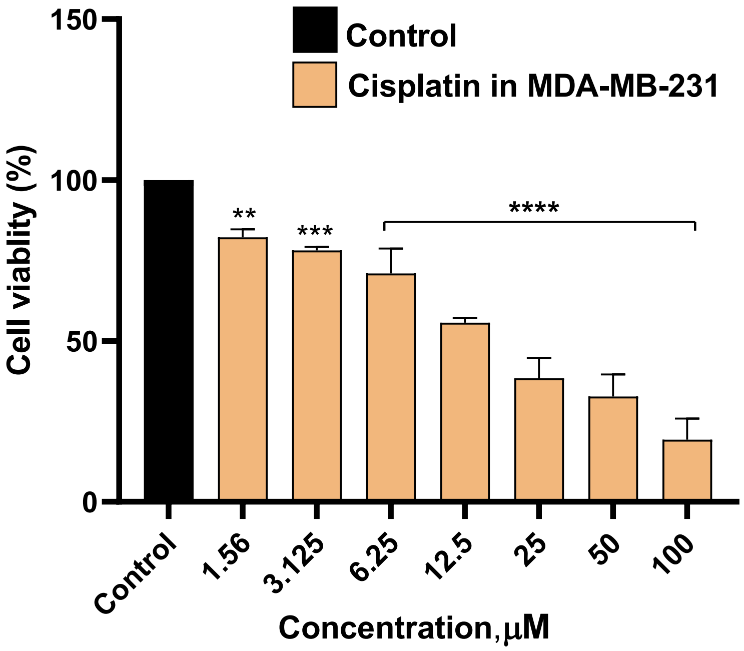

Figure S7. The percentage cell viability vs concentrations of cisplatin.

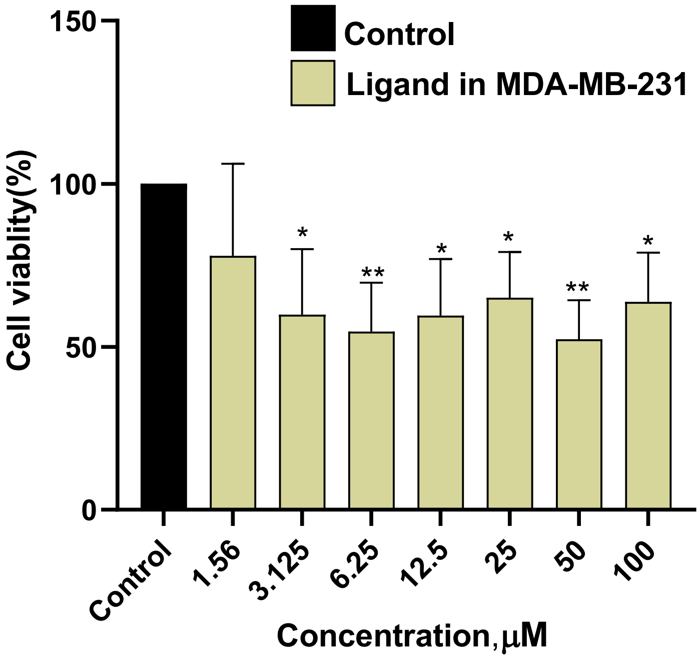

Figure S8. The percentage cell viability vs. concentrations of free ligand 1,5-bis-(salicylidene) thiocarbohydrazide.

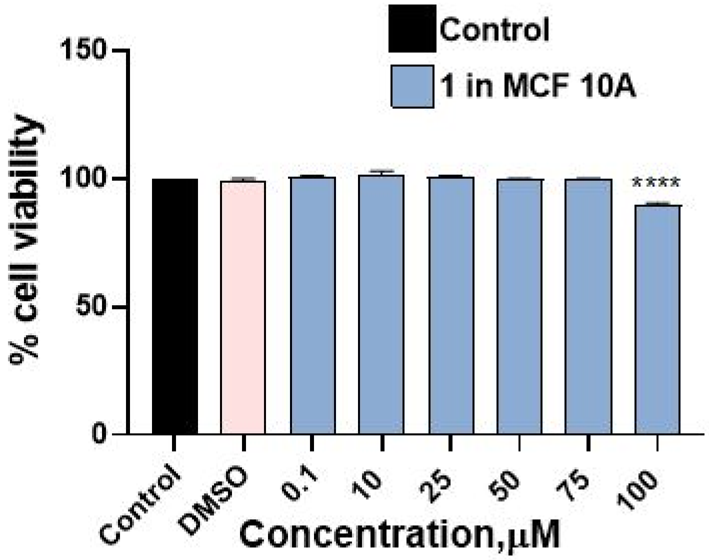

Figure S9. The percentage cell viability vs. concentrations of complex **1** (**K38**).
